# Targeting Prostate Cancer Metabolism Through Transcriptional and Epigenetic Modulation: A Multi-Target Approach to Therapeutic Innovation

**DOI:** 10.3390/ijms26136013

**Published:** 2025-06-23

**Authors:** Pedro Juan Espitia-Pérez, Lyda Marcela Espitia-Perez, Mario Negrette-Guzmán

**Affiliations:** 1 Facultad de Ciencias de la Salud, Laboratorio de Investigación Biomédica y Biología Molecular, Universidad del Sinú, Montería 230001, Colombia; lydaespitia@unisinu.edu.co; 2 Departamento de Ciencias Básicas, Escuela de Medicina, Universidad Industrial de Santander, Bucaramanga 680002, Colombia; maneguz@uis.edu.co

**Keywords:** metabolic reprogramming, prostate cancer, transcription factor, epigenetics, cancer treatment

## Abstract

Prostate cancer (PCa) therapy faces challenges due to tumor heterogeneity, plasticity, and progression. Metabolic reprogramming, a dynamic process, has emerged as a key focus in PCa treatment. However, conventional therapies targeting cancer-specific metabolic pathways or employing chemosensitizers are often limited by compensatory mechanisms and metabolic complexity. This review highlights the roles of transcription factors, including AR, p53, c-Myc, HIF-1, Nrf2, and PPARγ, in regulating PCa metabolism by influencing signaling pathways, enzymes, and gene expression. Multi-target compounds, particularly natural products, show potential for disrupting multiple metabolic enzymes, opening up new research possibilities. Notable examples include β-elemene, juglone, tannic acid, and withaferin A, which target critical metabolic processes through enzyme inhibition, transcription factor modulation, epigenetic changes, and protein interaction disruption. Naturally derived metabolites can elicit transversal responses in diverse metabolic pathways, particularly in p53 and MYC transcription factors. Additionally, compounds such as pentacyclic terpenoids (ursolic acid with ursane skeleton), sulforaphane, and isothiocyanate-related moieties may induce metabolic and epigenetic changes through S-adenosyl methionine (SAM) and acetyl-CoA modulation, potentially affecting new areas of research through metabolic processes. We propose a cooperative crosstalk between metabolic reprogramming and transcription factors/epigenetic modulation in PCa. This approach holds potential for expanding PCa therapeutics and opening new avenues for research.

## 1. Introduction

Prostate cancer (PCa) is the second most diagnosed cancer and the fifth leading cause of cancer death among men worldwide, contributing with an estimated 14.1% new cancer cases and 6.8% of all cancer deaths in males in 2020 [1]. PCa incidence has increased over the last year in American men and constitutes a significant death cause worldwide [2,3], particularly in low-income countries [4]. When a patient is diagnosed with PCa, the therapy mostly depends on the stage of the disease. At the initial stages, prostate tumors rely on androgen receptor (AR) signaling, and treatment therefore usually consists of radical prostatectomy, radiation, or pharmacological androgen deprivation therapies. However, when PCa becomes metastatic, or even castration-resistant (CRPC), the patient survival rate decreases [5]. Treatments against metastatic PCa possess a therapeutic threshold, and despite current and promising therapies, there is no clinically proven cure for CRPC [6,7].

The bottleneck in PCa therapy is compounded by the limited availability of clinically approved treatments, a global tendency in cancer research creating heightened pressure for the discovery of novel and improved drugs, as well as new treatment regimens [8]. The current traditional method of experimental “hit discovery” employed by R&D in pharmaceutical and phytopharmaceutical sectors needs reevaluation, especially due to the time-consuming process of target identification [9,10]. In addition, one must consider not only the effects on the target but also the “off-target” effects, which in many cases lead to adverse effects in vivo that contrast with the desired therapeutic effect [8].

During disease progression, prostate cancer cells undergo a series of metabolic changes to provide energy, biosynthesis, and redox requirements to support tumor growth [11,12]. This is known as “metabolic reprogramming”, a highly dynamic process that allows cancer cells to adapt to the hostile tumor microenvironment by altering glucose, lipid, and amino acid metabolism to suit their needs [12]. Metabolic reprogramming is influenced by a multitude of factors, including oncogenes, growth factors, hypoxia-induced factors, and the malfunction of tumor suppressor genes [13]. Besides tumor growth, cancer metabolic reprogramming promotes other critical processes during tumorigenesis, such as survival, invasion, metastasis, therapy resistance, and other pivotal cellular functions [14]. As tumorigenesis advances, cancer cells accrue additional mutations and alterations that further enhance metabolic reprogramming, thus accelerating tumor growth, proliferation, and progression [14]. This underscores an urgent need to escalate research focused on strategies to disrupt the inherent metabolic reprogramming of PCa.

To effectively target PCa from a metabolic perspective, it is crucial to focus not only on the enzymes involved in the process, but also take a holistic approach. The metabolic reprogramming of PCa produces metabolites that act as epigenetic modulators, including acetyl CoA, S-adenosyl methionine (SAM), and lactate. This mutual process benefits the cancer cell, as the same metabolic reprogramming of PCa supports and perpetuates epigenetic modulation. Moreover, the transcriptomic processes in PCa impact metabolic reprogramming by regulating enzyme expression, indirectly generating changes in the epigenetic landscape. This is also a two-way process, as the expression of specific transcription factors also requires epigenetic regulation.

This review delves into the possibility of inducing a form of metabolic reprogramming that can lead to a phenotypic reversal in PCa. By investigating the pharmacological strategies in PCa treatment, the goal is to address the essential question: what kind of compounds have proven (evidenced) a “multi-target” disruption in the inherent metabolic reprogramming of PCa? This literature review covers peer-reviewed articles from PubMed, Scopus, and Web of Science, focusing on metabolic reprogramming, deregulated transcription factors, and epigenetic-related modifications. The object is to identify potential chemotherapy candidates, particularly those described as multi-target inhibitors with transcriptomic or epigenetic effects. This work examines small molecules, drugs, active principles, and naturally derived compounds, emphasizing the inclusion of repurposing candidates when a favorable multi-target response is evident. Each article has been critically appraised to synthesize current knowledge and pinpoint research gaps. Additionally, analyses of literature reviews from other authors are included to reinforce certain aspects or ideas in the text, thereby enriching the discussion.

**Figure 1:**
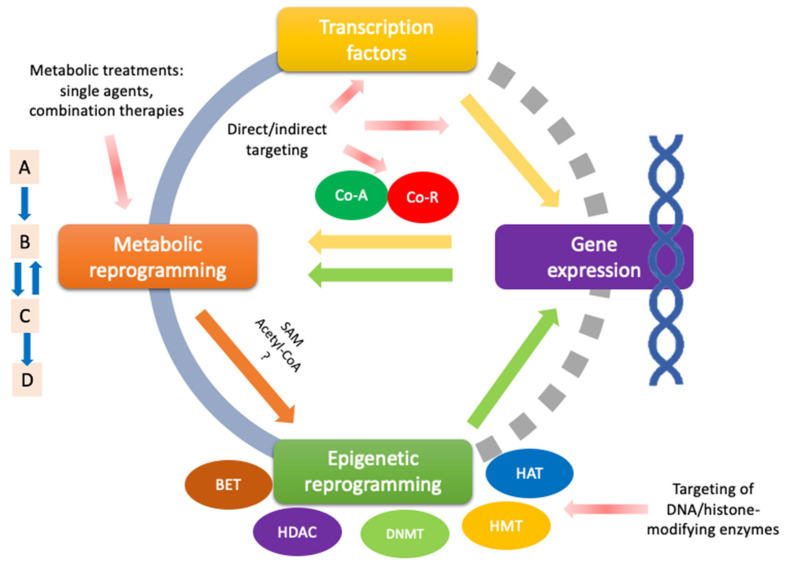
Schematic representation of the coordination of metabolic reprogramming and gene expression applied in the context of prostate cancer treatment. The classic approach of metabolic reprogramming-based treatment involves direct interference of components of the cancer-activated metabolic pathway. However, certain compounds can modulate epigenetic reprogramming via S-adenosylmethionine (SAM) and acetyl-CoA. The true extent of other metabolites that might modulate epigenetic reprogramming in PCa represents exciting research opportunities. Transcriptomic control uses indirect or direct targeting methods to disrupt the transcription factor or affect its transcriptional process. On the other hand, there is direct epigenetic control, and therapies are based on targeting DNA/histone modifying enzymes, affecting metabolic processes. Nevertheless, gaps in understanding transcription factors and epigenetic processes in PCa still need further research (gray dashed semicircle). Colored arrows show the crosstalk between cellular mechanisms. Red arrows show targeted cellular mechanisms where multi-target compounds might be most effective. Co-A (green) represents co-activators; Co-R (red circle) represents co-repressors. BET (bromodomain and extra-terminal domain proteins); HDAC (histone deacetylase); DNMT (DNA methyltransferase); HMT (histone methyltransferase); HAT (histone acetyltransferase).

Based on the literature review, this paper suggests a dynamic cooperative interaction between metabolic reprogramming processes, transcription factors, and epigenetics. This interaction could be utilized through pharmacological strategies to expand the potential of existing and promising therapies and advance research in this area (see Figure 1). In contrast to traditional one-hit enzyme-directed compounds or combination therapies to increase chemotherapy effectiveness, the transcriptomic-directed and epigenetic reprogramming compounds can potentially have multi-target effects in PCa. This study analyzes the main disrupted transcriptomic pathways with metabolic reprogramming effects, followed by the signaling pathways that act as metabolic activators supporting these transcriptomic effects, and then presents a group of compounds that can induce multi-target response and partially reverse the phenotypic metabolic traits in PCa. A discussion of how multi-target compounds may act is provided in the text, focusing on affected signaling cascades, disrupted transcriptomic regulation, and metabolic epigenetic regulation as important therapeutic approaches for PCa. Evidence from in vitro and in vivo experiments is supported through the assay from a transcriptomic and epigenetic perspective. Furthermore, a dedicated section reviews the clinical trials involving several of the promising compounds previously discussed. The last sections will summarize and discuss the main findings, addressing future directions and research gaps.

## 2. Metabolic Reprogramming-Based Treatments for Prostate Cancer: The Classic and the Chemosensitization Approaches

PCa cells undergo significant metabolic reprogramming, adapting processes like citrate and zinc metabolism, glucose utilization, lipid metabolism, and glutamine and one-carbon (1C) metabolism to support their growth and survival. In the normal peripheral zone of the prostate gland, high levels of citrate are maintained through the inhibition of aconitase (ACO2) by zinc, preventing citrate oxidation in the Krebs cycle [15,16,17]. However, in PCa cells, this balance is disrupted, leading to decreased zinc levels, increased ACO2 activity, and citrate oxidation, which correlates with malignancy [18]. This metabolic shift is regulated by zinc transport proteins (ZIP and ZnT), where ZIP transporters are commonly downregulated, while specific ZnT transporters are upregulated, ultimately contributing to zinc depletion in malignant cells [19].

Citrate oxidation leads to citrate-deficient PCa cells. Logically, the pharmacological approach of using citrate has been tested previously to reverse the malignant phenotype of PCa. It seems that citrate effects on PCa metabolic reprogramming are directed towards fructose-6-phosphate-1 kinase (PFK1) inhibition [20] and other biological effects such as a “pro-death” autophagy pathway [21]. Metabolic reprogramming abilities of citrate in PCa might be dose-dependent. Cytosolic citrate levels above 10 mM are necessary for PFK1 inhibition, but chronic citrate exposure increases PCa resistance. PC-3 cells exposed to chronic citrate showed increased PFK1 expression and more resistance to regular treatments [20]. Using citrate at controlled doses from 10 mM seems to generate PFK1 inhibition and other effects, such as apoptosis induction in several cancer cell lines [22]. Further impacts of citrate treatment in PCa cells, such as inhibition of other glycolytic enzymes, might need additional research [23].

PCa cells exhibit increased glucose metabolism similar to other tumors, with enhanced glucose transporter expression (GLUT1, GLUT5, GLUT12) and glycolytic enzymes (HK1/2, PFKB2), regardless of androgen receptor (AR) signaling [24,25,26,27,28,29,30]. Early-stage PCa cells favor fructose metabolism, while advanced stages and CRPC rely more on lipid metabolism and glycolysis, characterized by high expression of monocarboxylate transporters (MCT), HK2, and pyruvate dehydrogenase kinase (PDK1) [31]. Lipid metabolism plays a crucial role, with PCa cells utilizing fatty acid (FA) β-oxidation for energy and overexpressing enzymes involved in FA and cholesterol synthesis under AR control, such as ATP-citrate lyase (ACLY), acetyl-CoA carboxylase (ACC), fatty acid synthase (FASN), stearoyl-CoA desaturase-1 (SCD1), ELOVL fatty acid elongase 5 (ELOVL5), 3-hydroxy-3-methylglutaryl-CoA reductase (HMGCR), and squalene epoxidase (SQLE) [16,32]. Sterol regulatory element-binding proteins (SREBPs) regulate these pathways, with SREBP1 involved in FA synthesis and SREBP2 in cholesterol synthesis via the mevalonate pathway [33].

Glutamine metabolism is vital for PCa cells, involving transporters from the solute carrier (SLC) family (EAAT3, ASCT1, ASCT2) [34] and the conversion of glutamine to glutamate and subsequently to α-ketoglutarate (α-KG) for the tricarboxylic acid (TCA) cycle and glutathione synthesis. Increased expression of glutaminase (GLS1) is noted in metastatic castration-resistant prostate cancer (mCRPC) [35]. The 1C metabolic pathway, involving enzymes like serine hydroxymethyltransferase (SHMT), methylenetetrahydrofolate dehydrogenase (MTHFD), and methionine synthase (MS), is crucial for synthesis of nucleotides, amino acids, and SAM, a key methyl donor for epigenetic regulation [36]. The pathway’s compartmentalization and the high prevalence of genetic polymorphisms in enzymes like methylenetetrahydrofolate reductase (MTHFR) suggest its importance in PCa progression [37].

These disrupted metabolic pathways in PCa cells are controlled by genomic alterations, hypoxia, and interactions with stromal cells in the tumor microenvironment (TME) [16,30]. Genetic mutations in androgen signaling, DNA repair, and PI3K signaling genes (*AR*, *ATM*, *TP53*, *FOXA1*) are common, with further alterations like MYC overexpression, *PTEN* inactivation, and *TMPRSS2-ERG* fusion driving PCa progression [30,38]. Epigenetic regulators like *EZH2* also modulate glycolysis in metastatic PCa, highlighting the dynamic and complex nature of metabolic reprogramming in PCa [39].

The use of therapy targeted towards cancer metabolism relies on the suppression of a specific pathway considered “cancer-promoter”. This entails blocking an enzyme or interfering with a specific signaling pathway to elicit a comprehensive impact on a targeted metabolic pathway. This approach could be deemed “classic” as it has been predominant in recent years, focusing on identifying hyperactivated metabolic pathways, regulators, and essential enzymes in PCa metabolic rewiring [40]. Therefore, often, these compounds are referred to as “inhibitors”. Specific molecular responses involve the alteration of metabolic pathways during treatment administration. The latter can be observed through decreased enzyme activity or the dysregulation of a pathway leading to metabolite modulation, which consequently affects other cell processes such as epigenetic regulation; this could explain some findings, such as the apparent transcriptional disruption following metabolic therapies [41]. However, there is some caution regarding the use of classic metabolic inhibitors. Despite their promise, it is necessary to note that there are not many approved inhibitors or metabolic treatments for clinical use, and many currently known in the literature are in a preclinical context [30,42], and a narrow therapeutic window characterizes the use of these compounds [16,30,42]. Additionally, it is noteworthy that many enzymes targeted by metabolic treatments are also essential for normal cells, which explains the inherent toxicity of these compounds and limits their use due to side effects [42]. As monotherapies, several natural, synthetic, and small molecules with metabolic inhibitor activity can be fully addressed in Table 1 in the context of experimental treatments for PCa.

Metabolic reprogramming-based combination treatments and their targets are summarized in Table 2. These are called “chemosensitizers” since they make a cancer cell more sensitive to chemotherapy. Combining two or more targeted anticancer agents can overcome resistance, enhance the response to monotherapy drugs, reduce dose-limiting single-agent toxicity, and expand the range of treatment options [43,44,45]. Some studies shown in this review focused on combined treatments for PCa with a specific metabolic inhibitor and an approved chemotherapeutic, such as enzalutamide, abiraterone or docetaxel.

One of the main explored mechanisms in dual-hit strategies or combinational approaches is the carnitine palmitoyltransferase 1 A (CPT1A) inhibition. A decreased CPT1A expression is associated with decreased AKT content and activation, likely driven by a breakdown of membrane phospholipids and activation of the inositol polyphosphate-5-phosphatase K (INPP5K). This results in increased AR action, which may be exploited with an AR-inhibitor [46]. In other cases, CPT1A inhibitors may serve as chemosensitizers, either in combination with another metabolic drug targeting a lipid-related enzyme or alongside a specific chemotherapeutic agent (Table 2).

Metabolic reprogramming effects of one drug, valproic acid (VA), are promising in the field of mCRPC treatment. The combination of VA, simvastatin and docetaxel decreased the expression of the yes-associated protein (YAP), an oncogen [47]. Effects of VA were potentiated with simvastatin, an HMG-CoA reductase inhibitor. Metabolic reprogramming included an increased AMP-activated kinase phosphorylation. However, synergistic interaction was obtained with VA-simvastatin-docetaxel combination, and these findings were replicable both in vitro and in vivo (Table 2). Extensive research supports the metabolic reprogramming capabilities of VA, a histone deacetylase (HDAC) inhibitor [48,49,50,51,52], but other evidence demonstrates neurodifferentiation induced phenotypes which probably explain the renounce of using VA for PCa treatment [53].

**Table 1:** **Metabolic reprogramming strategies for PCa based on single targeting.** In some cases, one molecule defined for a single target elicited a multiple response.

Metabolic Reprogramming Mechanism	Target	Drug	Experimental Model	Methods	Main Results	Reference
**Citrate metabolism via Krebs Cycle**	ACO2	Camptothecin	LNCaP and PC3 cell lines	WB and mitochondrial enzymatic activity assay	Upregulation of p53, decreased ACO2 expression	[54]
**Zinc metabolism regulation**	ZIP	5-azacytidine	RWPE-1 normal prostate epithelium cell line; LNCaP and DU-145 prostate carcinoma cell lines	qRT-PCR, chromatin immunoprecipitation (ChIP)	Reduced cell growth, reactivation of silenced ZIP1 and ZIP3	[55]
**Glucose metabolism**	GLUTs inhibitors	Genistein, phloretin, apigenin, and daidzein	PNT1A normal prostate cells, LNCaP and PC3 cells, LNCaP and PC3 androgen insensitive and androgen sensitive clones	WB, cellular location confirmation by ICC	Apigenin and phloretin modified GLUT1 and GLUT4 expression, reducing cell proliferation in androgen independent PCa cells	[56]
	PFK1	Citrate	PC3, LNCaP and WPMY-1 cell lines	Cell proliferation assays, apoptosis determination by Annexin-V-FITC/PI Double Staining; WB	Citrate triggers autophagic cell death in prostate cancer cells by inhibiting the CaMKII/AKT/mTOR pathway, which may be linked to reduced PFK1 activity	[22]
**Lipid metabolism**	FASN	TVB-2640	LNCaP and C4-2 prostate carcinoma cell lines	WB for protein confirmation. STRING, GSEA, RNA-Seq, and K-M analyses using database search for FASN expression and correlations with clinical parameters	Increases Lipin1 and ASCL1 expression, both FASN interactors, with increased lipid accumulation. Cell cycle arrest	[57]
		TVB-3166	22Rv1 cell line	β-Tubulin Confocal Immunofluorescence, WB	Reduced β-tubulin mRNA expression	[58]
		IPI-9119	LNCaP, LNCaP-95, 22Rv1, C4-2, xenograft implant, and MSK-PCa3 organoids (advanced mCRPC)	Real time qPCR, RNA-Seq and metabolomic profiling	Decreased cyclin A2 expression, down-regulation of pathways associated with amino acid and protein translation (LNCaP), and purine and pyrimidine synthesis (22Rv1 and LNCaP 95). Reduced expression and transcriptional activity of AR-FL and AR-V7	[59]
		Triclosan	Panel of prostate cancer cells (LNCaP, C4-2B, PC-3, 22RV1, RWPE-1 LAPC4, BPH-1, WPMY-1, and 3T3 cells)	Live imaging, qRT-PCR, WB	Increased cytotoxicity in PCa cell lines. Significant reduction of FASN gene expression, increased PLA2G6 expression, reduced lipid content in LNCaP cell lines	[60]
	ACLY	miR-22	RWPE-1 normal prostate epithelium cell line and PC3 PCa cell lines	qRT-PCR, WB	Disrupted ACLY post transcriptional control, decreased cell proliferation and invasion, downregulation of FASN and HMGCR	[61]
	ACC	Soraphen A	LNCaP, PC3-M (PC-3M-luc-C6), and BPH-1 cells	Fatty acid oxidation measurement, acetate incorporation assay, WBWestern blot	At nanomolar concentrations, soraphen A blocks fatty acid synthesis, stimulating fatty acid oxidation in LNCaP and PC-3M cells	[62]
		PF-05175157	Prostate-derived explants, LNCaP cell line	Metabolomic profiling (MALDI-MSI), RNA-Seq, IHC	Reduced fatty acid elongation, decreased expression of Ki67, cleaved caspase-3, and increased p-ACC expression in LNCaP cells	[63]
	SCD1	BZ36	PNT2 prostate epithelial cell line, LNCaP and C4-2 cell lines, human prostate tissue samples, xenograph implant	qRT-PCR, WB, IHC	Decreased de novo lipogenesis, AKT/PIP3 and GSK3α/β/β-catenin signaling pathways, cell growth arrest	[64]
	SREBP	Fatostatin	LNCaP and C4-2B cell lines, and xenograph implant	qRT-PCR, WB, IHC	Inhibition of SREBP processing and transcriptional activity, decreased Ki67 expression and increased cleaved PARP	[65]
	Production of chemokines CCL2, CXCL12, receptor CXCR4, and pro-inflammatory cytokines TNF-α and IFN-γ, pro angiogenic regulators (VEGF, CXCL8, angiogenin) and MMP-9	ALCAR	In vitro: PC-3, DU-145, LNCaP, 22Rv1 and benign prostate hyperplasia cell line (BPH); In vivo xenograph model	Flow cytometry, WB	Reduced expression of VEGF, CXCL8, CCL2, angiogenin and metalloprotease MMP-9. Inhibited expression of CXCR4, CXCR1, CXCR2 and CCR2	[66]
	ACSL1, ACC, ACeCS1, FASN, Lipin 1	Tannic acid	C4− 2, DU145 and PC-3 cells	RNA extraction and mRNA microarrays, bioinformatic iPathway guide analysis	Induced ROS and endoplasmic reticulum (ER) stress, nuclear disorganization, apoptosis	[67]
	ACC, ACLY, FASN, CPT1A	Withaferin A	LNCaP and 22Rv1 cell lines	RNA-Seq, KEGG pathway analysis, qRT-PCR, xenograph implant	Downregulation of ACC, ACLY, FASN, CPT1A	[68]
**Glutaminolysis**	GLS1/2	CB-839	PC3 and PC3M	Reverse-phase protein array (RPPA) and WB	Metabolic differences in metastatic cell line, including increased glutamine utilization corroborated by differences in levels of phosphorylated AKT (pS473P) and mTOR in PC3M	[69]
			LNCaP, PC3, enzalutamide-resistant C4-2MDVR cells	Tissue microarrays and IHC for biomarker validation, qPCR for transcript validation, LC/MS for metabolite assessment	GLS1 up regulated by AR signaling and glutaminase C (GAC) activation. Pharmacological inhibition of GAC show better treatment effect for castration resistant PCa	[35]
	AR	Proxalutamide	AR-positive (22RV1 and LNCaP) and AR-negative cells (PC3 and DU145)	LC-Q/TOF-MS for metabolite assessment	Inhibition of glutamine metabolism, redox homeostasis, and de novo pyrimidine synthesis in AR-positive PCa cells	[70]
**Mitochondria and oxidative stress**	Mitochondria	Synthetic non-glycoside analogs from sugar conjugates of 1,4-naphthoquinone urchin pigments spinochromes	PC-3, DU145, 22Rv1, and LNCaP, as well as human prostate non-cancer cell lines RWPE-1 and PNT2	Apoptosis determination by Annexin-V-FITC/PI Double Staining; WB, Tandem Mass Spectrometry, Bioinformatic Ingenuity Pathway Analysis (IPA)	Mitochondria membrane permeabilization, ROS upregulation and release of cytotoxic mitochondrial proteins (AIF and cytochrome C), apoptosis	[71]

AR: androgen receptor; GLS1/2: glutaminase isoform 1 and 2; ASCL1: achaete-scute homolog 1; ACC: acetyl CoA carboxylase; pACC: phosphorylated form of acetyl CoA carboxylase; ACeCS1: acetyl-CoA synthetase; FASN: fatty acid synthase; CPT1A: carnitine palmitoyl transferase 1 A; SREBP: sterol response element binding protein; SCD1: stearoyl-CoA desaturase; ACLY: ATP-citrate lyase; GLUTs: glucose transporters; ACO2: mitochondrial aconitase; p53: protein 53 kDa; qRT-PCR: quantitative reverse transcription polymerase chain reaction; CaMKII/AKT/mTOR: Ca^2+^/calmodulin-dependent protein kinase II (CaMKII)—protein kinase B (AKT)—mechanistic target of rapamycin (mTOR) pathway; STRING: functional protein association networks; GSEA: gene set enrichment analysis; KEGG: Kyoto Encyclopedia of Genes and Genomes pathway analysis; RNA-Seq: RNA sequencing; PLA2G6: phospholipase A2 of group VI; ZIP1 and ZIP3: zinc-regulated, Iron-regulated transporter-like proteins 1 and 3; AR-FL: androgen receptor full length; AR-V7: androgen receptor splice variant 7; HMGCR: hydroxy-methyl-glutaryl-CoA; PIP3: phosphatidylinositol (3,4,5)-trisphosphate; GSK3α/β: glycogen synthase kinase 3; Ki67: antigen Ki67; PARP: poly (ADP-ribose) polymerases; CCL2: chemokine CCL2; CXCL 12: chemokine CXCL 12; CXCR4: chemokine receptor C-X-C chemokine receptor type 4; TNF-α: tumor necrosis factor alpha; IFN-γ: gamma interferon, VEGF: vascular endothelial growth factor, CXCL8: C-X-C motif chemokine ligand 8; MMP9: matrix metalloproteinase 9; ALCAR: acetyl-L-carnitine; MALDI-MSI: matrix assisted laser desorption/ionization mass spectrometry imaging; ICC: immunocytochemistry; IHC: immunohistochemistry; K-M: Kaplan-Meier; LC/MS: liquid chromatography/mass spectrometry; LC-Q/TOF-MS: liquid chromatography-quadrupole time-of-flight tandem mass spectrometry; FITC/PI: fluorescein isothiocyanate/propidium iodide double staining; WB: Western Blot.

**Table 2:** Combination treatments for metabolic reprogramming in prostate cancer.

Metabolic Pathway	Treatment	Target	Experimental Model	Methods	Main Results	Reference
**Lipid metabolism**	miR-33a plus statins decreasing CPT1A and HADHB	CPT1A and HADHB	LNCaP and VCaP cells	MicroRNA transfection, cell proliferation test, Matrigel invasion test, soft agar colony test	Decreased cellular progression	[72]
	Combinations of BMS-303141 and SB-204990 with enzalutamide	ACLY	C4-2, LNCaP and LNCaP abl (long-term androgen-free incubation), PC3 and 3T3-L1, focused on CRPC model. Combination treatments with ACLY inhibitor, BMS-303141 (ACLYi)	GS-MS metabolite measurements, qRT-PCR, immunoblotting, RNA-Seq	ACLYi and enzalutamide suppresses AR target gene expression in DHT treated and androgen depleted cells	[73]
	TVB-3166 and paclitaxel	FASN	22Rv1	β-Tubulin Confocal Immunofluorescence, WB	Reduced b-tubulin mRNA expression	[58]
	Etomoxir, perhexiline, ranolazine and enzalutamide	CPT1A	22Rv1, LNCaP-MDV resistant and TRAMPC1 cells	RNA-Seq, RT PCR, CalcuSyn for CI determination, and xenograph implant	AR-related genes upregulation due to CPT1A KD. Decreased tumor growth with enzalutamide combinations	[46]
	Perhexiline and AUY922 (HSP90 inhibitor)	HSP90	Patient derived explants, LNCaP, C4-2B, and 22RV1 cell lines	MS, flow cytometry and qRT-PCR	Increased protein expression of fatty acid oxidation and oxidative phosphorylation pathways following AUY922 treatment. Increased cell cycle arrest and apoptosis following cotreatment with perhexiline	[74]
**Glutaminolysis**	CB-839 with talazoparib (PARP inhibitor)	GSL1	DU145 cell line	CalcuSyn for CI determination, flow cytometry, immunofluorescence, xenograph implant	Synergistic effects of CB-839 and talazoparib, cell growth inhibition, decreased tumor volume. No results for metabolic reprogramming	[75]
**Metabolic-related pathways with cellular signaling modulation**	Valproic acid and simvastatin, in combination with docetaxel	Mevalonate pathway and AMPK	PC3, 22Rv1, DU145, DU145R80, and LNCaP. Creation of a 22Rv1 docetaxel-resistant cell line	CalcuSyn for CI determination, clonogenic agarassay, WB, RT-PCR, spheroid forming assay, and xenograph implant	Valproic acid and simvastatin combination inhibited YAP oncogene activity in a mCRPC model, increased AMPK fosforilation and downstream HMG-CoA reductase inhibitory phosphorylation. Synergistic potentiated effects with docetaxel.	[47]

RT-PCR: reverse transcription polymerase chain reaction; AMPK: AMP-activated protein kinase; GLS1: glutaminase isoform 1; HSP90: heat shock protein 90; CPT1A: carnitine palmitoyl transferase 1 A; ACLY: ATP-citrate lyase; HADHB: hydroxyacyl-CoA dehydrogenase trifunctional multienzyme complex subunit beta; CI: combination index; YAP: yes-associated protein; qRT-PCR: quantitative reverse transcription polymerase chain reaction; RNA-Seq: RNA sequencing; GS/MS: gas chromatography coupled to mass spectrometry; KD: knock-down; WB: Western Blot.

## 3. The Crosstalk Between Transcription and Epigenetics to Influence Metabolic Reprogramming in Prostate Cancer

Recent studies have revealed an increasingly prevalent trend towards observing metabolic reprogramming processes in light of cellular plasticity [76,77]. Thus, metabolic reprogramming exhibits an even more dynamic nature, governed by a circuit of cooperation relationship between cellular metabolism and gene transcription [42,78]. Consequently, PCa can adequately coordinate signaling pathways during initiation and progression phases, modulate nutrient availability, condition the TME, and gradually respond with a gene expression program tailored to its metabolic needs [78]. To enact this gene expression program, PCa must finely adjust the expression of metabolic genes either through epigenetic modifications or transcriptional regulation [42]. In this latter aspect, transcription factors serve as the regulatory interface for cancer research, playing an indispensable role in metabolic reprogramming.

**Figure 2:**
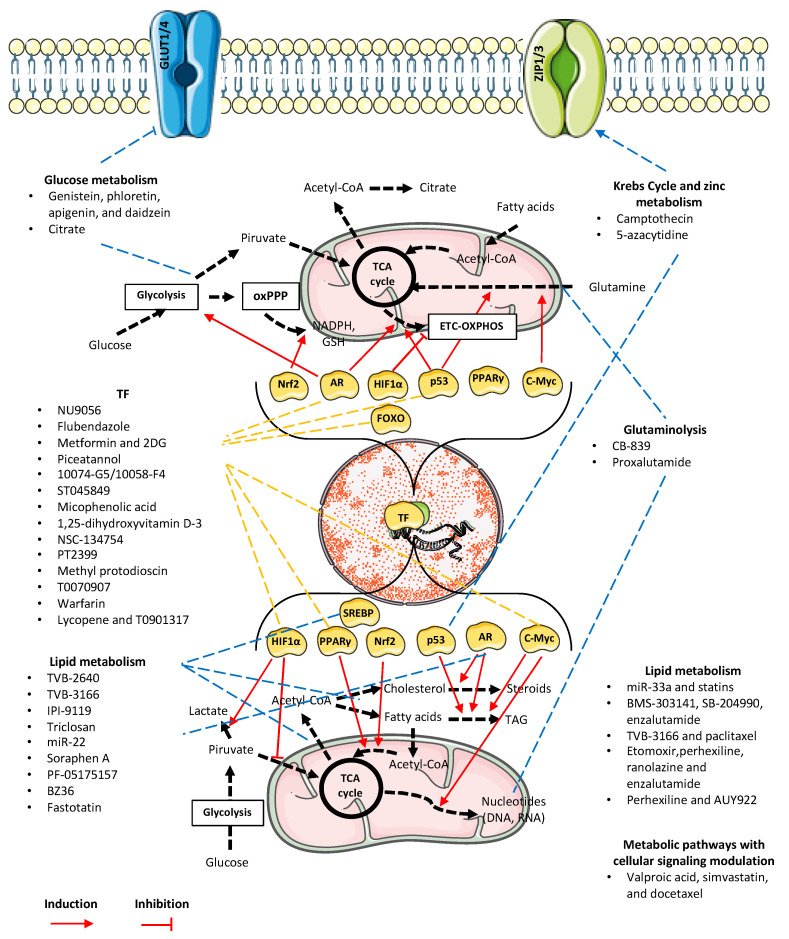
Overview of key metabolic pathways, transcriptomic modulation, and chemotherapeutic and metabolic compounds for prostate cancer treatment. This figure summarizes treatments discussed throughout this review and illustrates the interplay between metabolic reprogramming and major regulatory signaling pathways in prostate cancer cells, highlighting potential therapeutic targets. Key metabolic substrates include glucose, glutamine, fatty acids, lactate, pyruvate, cholesterol, triacylglycerol (TAG), steroids, and citrate. The main metabolic pathways represented are glycolysis, glutaminolysis, the tricarboxylic acid (TCA) or Krebs cycle, electron transport chain and oxidative phosphorylation (ETC-OXPHOS), the oxidative branch of the pentose phosphate pathway (oxPPP), and lipid metabolism. Relevant metabolic enzymes and transporters are shown, including glucose transporters (GLUT1/4), zinc transporters (ZIP1/3), and sterol regulatory element-binding protein (SREBP), along with their roles in regulating nutrient flux and metabolite availability. The generation of key metabolic intermediates—such as acetyl-CoA, NADPH, and reduced glutathione (GSH)—is also indicated. Red arrows illustrate the convergence between gene regulatory networks and metabolic pathways. Color-coded compounds represent different therapeutic strategies: blue indicates classical single-target metabolic agents; purple denotes combinatorial treatments; and yellow highlights compounds with demonstrated transcriptomic modulation (TF). Dashed lines in each color reflect the principal molecular mechanisms affected by the corresponding treatment.

In a recent review, the term “dysregulated transcription factors” was used to express those transcriptionally acting proteins that had a relationship with metabolic action in cancer [42]. The transcription factors with probable metabolic correlation with PCa, including AR, are protein 53 kDa (p53), cellular myelocytomatosis protein (c-Myc or MYC), hypoxia-inducible factor 1 (HIF-1), and nuclear factor erythroid 2-related factor 2 (Nrf2). However, one transcription factor, peroxisome proliferator-activated receptor gamma (PPARγ) has an important and underestimated role in PCa. The following sections will show evidence of how dysregulation of specific transcription factors, their histone-modifying coactivators and corepressors, and their subsequent modulation can eventually induce and potentiate metabolic reprogramming responses in PCa. A compilation of studies comprising the use of biomarkers and therapeutic approaches is also described (Table 3). The plasticity of transcriptomic modulation plays a critical role in driving metabolic reprogramming. Figure 2 illustrates the dynamic interplay between metabolic pathways and key regulatory signaling networks in prostate cancer cells, highlighting potential therapeutic targets and the treatments discussed throughout this review.

### 3.1. AR

AR is a transcription factor responsive to androgens, belonging to the family of nuclear hormone receptors. AR contains four functional domains, including the N-terminal domain (NTD), DNA-binding domain (DBD), hinge, and ligand-binding domain (LBD) [110]. In the absence of ligand, AR remains inactive but highly receptive in the cytoplasm, comprising a dynamic heterocomplex composed of heat shock proteins (HSP), co-chaperones, and proteins containing tetratricopeptide repeats (TPR) [111]. However, coactivator recruitment begins once AR encounters ligands such as testosterone or dihydrotestosterone (DHT). AR translocates to the nucleus to activate the androgen response element (AnRE) and increase the transcription of AR genes [110].

The activity of AR as a nuclear receptor is mediated by coactivators and corepressors that control acetylation and deacetylation. We find histone acetyltransferases (HATs) such as Tip60, p300, and p300/cAMP response element-binding protein-associated factor (P/CAF) for acetylation [110]. Tip60 is highly important in stabilizing AR as it interacts with the LBD [112]. On the other hand, we have histone deacetylases (HDACs) such as HDAC1 and Sirtuin 1, with the upregulation of HDAC1 leading to a reduction in AR activity [110]. Notably, the interaction of Tip60 is crucial for AR, which has been exploited in therapeutic alternatives to destabilize AR in the context of PCa [79,110] (see Table 3). Studies on modulating HAT activity for AR signaling are an important new target for therapies that require future research.

The transcription factor AR controls the citrate oxidation via zinc transporter ZIP1 expression. Modulation of zinc levels is necessary in normal prostate, but it is lost in PCa due to the activation of mitochondrial aconitase by AR [113]. Additionally, AR is a modulator of glucose metabolism, although this is only sometimes the case. Androgens increase the expression of GLUTs and, in some cases, due to the homology of the LBD of AR with that of GLUT1, there is a possibility that androgens may directly bind to GLUT1, modulating cellular glucose intake directly [114]. Likewise, GLUTs are regulated by metabolic regulators such as AMP-activated protein kinase (AMPK) [26]. Other glycolytic enzymes AR activates include HK1/2, PFK, and PFKFB2 [31,110]. The relationship between these enzymes and metabolic regulators is determined by AR, generating a hybrid phenotype of glycolysis/OXPHOS in PCa through multiple means, inducing glycolysis, either by the activation of PFKFB2 and HK2, or the transport of pyruvate to the mitochondria mediated by dynamin-related protein 1 (DRP1) or of OXPHOS via the AMPK-peroxisome proliferator-activated receptor gamma coactivator 1-alpha (PGC1α) signaling cascade [16,31]. AMPK stimulates carbohydrate use, while PGC1α stimulates mitochondrial biogenesis.

Regarding the association between AR and lipid metabolism regulation in PCa, we had previously described that AR sustains the lipogenic metabolic program by upstream regulation of SREBPs, which coordinate the subsequent gene induction of ACC, ACLY, FASN, and HMGCR [115,116]. Another essential process mediated by AR is cholesterol import, a step towards the steroidogenic mechanism of PCa. AR regulates the expression of scavenger receptor-related Protein 8 (LRP8), scavenger receptor class B member 1 (SCARB1), and the low-density lipoprotein receptor (LDLR) [117]. The mechanisms mentioned above ensure metastatic phenotypes of PCa. Increased aggressiveness of PCa and progression to androgen-resistant phenotypes, such as CPRC, which regain AR signaling and its transcribed genes, are related to specific mutations and even induction of splice variants (alternative splicing, or AS) [16].

The increased expression of the ASCT2 glutamine transporter and GSL1 have been associated with an increased AR, MYC, and mechanistic “mammalian” target of rapamycin (mTOR) pathways’ activation, to ensure PCa progression, since glutamine is an mTOR effector. A study focused on ASCT2 inhibition as a PCa therapeutic strategy [118].

### 3.2. p53

p53 is a DNA-binding transcription factor controlling genes of cell-cycle checkpoints or programmed cell death (apoptosis) following exposure to ionizing radiation, ultraviolet (UV) light, or various other agents that cause damage to DNA [119]. Activation of p53 occurs through several mechanisms, including phosphorylation and dephosphorylation catalyzed by protein serine/threonine phosphatase-1, acetylation facilitated by the transcriptional coactivator family p300/CBP, and conformational alterations induced by the prolyl isomerase Pin1 [119]. Usually, p53 is a well-known tumor suppressor. However, mutations in the TP53 gene codifying p53 are the most frequent in human cancers comprising somatic and germline mutations and even TP53 polymorphisms [120].

Despite its cellular functions, p53 possesses other functions at the metabolic level. Loss of p53 leads to metabolic reprogramming, shifting from more OXPHOS-dependent phenotypes to glycolytic-dependent ones. In the case of PCa, a previous study showed that amino acid restriction, particularly glutamine, increases glucose consumption in the DU145 cell line modulated via p53 [121], which could relate glutaminolysis to p53. A study focused on an in vitro model found a feedback mechanism between inhibited p53 and the transcriptional activity of the solute carrier family seven-member 11 transporter, SLC7A11, which is responsible for glutamine movement [81]. Treatment with Flubendazole, an FDA-approved anthelmintic, elicits valid antitumor effects by increasing p53 expression, decreasing the transcription of SLC7A11, and promoting ferroptosis in CRPC [81] (see Table 3).

Lipid metabolism is an essential metabolic pathway for PCa, especially in advanced PCa. During progression, PCa cells show a marked inhibition of p53. The latter occurs through protein exonuclease 1 (EXO1), which has recombination capacity. EXO1 inhibits p53, activating SREBP1, and promotes lipogenesis in PCa, known as the p53-SREBP1 axis [122]. In the case of CRPC, the SREBP2 isoform is predominant in regulating steroidogenesis. PTEN/p53 deficiency makes this cancer dependent on cholesterol metabolism since this deficiency transcriptionally activates squalene epoxidase (SQLE) via activation of SREBP2 [84].

### 3.3. MYC

MYC, mostly referred as c-Myc, is known as a “super-transcription factor”, since the MYC oncoproteins (C-Myc, N-Myc, and L-Myc) controls the transcription of nearly 15% of expressed genes [123]. Normally, MYC combines with MAX (MYC/MAX), its co-effector, to create a heterodimer that attaches to DNA at the E-box region (CACGTG). This configuration primarily consists of the basic-region (BR), helix-loop-helix (HLH), and leucine-zipper (LZ), all essential for DNA binding. Accumulation of MYC at the promoter sequences of target genes can also augment the transcriptional activity of genes [123]. Curiously, the MYC/MAX complex is mostly expressed in LNCaP, an androgen-sensitive prostate cancer cell line derived from a lymph node metastasis, which represents an early-stage, less aggressive metastatic phenotype [124].

Through its adaptor, thioredoxin-interacting protein (TXNIP), c-Myc regulates aerobic glycolysis activity. This axis operates reciprocally, functioning as a switch promoting glutaminolysis via GLS1 in aggressive PCa [125]. The availability of non-glucose nutrients such as amino acids is exploited in PCa tumors thanks to c-Myc [126]. This regulation is driven by the loss of p62 in stroma, which is related to tumorigenesis through the mTORC1/c-Myc mechanism favoring tumor redox homeostasis and regulating glycolysis and amino acid metabolism [127]. The association between glucose and amino acid metabolism in PCa represents an intriguing pathway to explore due to its connection with c-Myc’s adaptor role in various glycolytic and glutaminolysis processes, which could provide new treatment alternatives for both early and advanced PCa. Indeed, MYC is a “driver amplification,” a genetic aberration that increases PCa plasticity and promotes its progression [128]. In advanced PCa and AR-inhibited phenotypes, the activity of DRP1 and MYC is increased, resulting in a glycolytic shift and resistance phenotypes in AR blockade therapy-resistant PCa [129].

The actions of AR, MYC, and mTOR downstream converge in increasing the expression of glutamine transporters SLC1A4 and ALC1A5 in PCa. C-Myc is a context-dependent regulator of transporters SLC1A4 and ALC1A5 [130].

Moreover, MYC is associated with dysregulated lipid metabolism in PCa [131], favoring an increase in de novo lipid synthesis through increased transcription of ACLY, ACC1, and FASN enzymes [132]. MYC activates apolipoprotein A-I (ApoA-I) in advanced PCa (metastatic or neuroendocrine prostate cancer, NEPC) [133].

MYC increases mitochondrial DNA levels in PCa precancerous lesions [134]. Surprisingly, it initiates a biogenesis process at the onset of this type of cancer progression and can also reactivate mitochondrial functionality in advanced PCa. In a previous study, it was found that HSP90 (mitochondrial chaperone) regulates CIpP (mitochondrial protease) expression via c-Myc, allowing their physical interaction (HSP90-CIpP). This process restores mitochondrial functions in advanced PCa [95] (see Table 3).

MYC is also associated with nucleotide metabolism in PCa. C-Myc is a known stimulator of de novo purine synthesis (XMP conversion regulated by c-Myc and independent of AR) [91].

### 3.4. HIF-1

The HIF-1 transcription factor is a heterodimer consisting of one of three hypoxia-induced alpha subunits (HIF-1α/2α/3α) and a beta subunit (HIF-1β, also known as aryl hydrocarbon receptor nuclear translocator (ARNT)) [135]. The most studied heterodimeric form is HIF-1α/HIF-1β, which activates HIF-1α under hypoxia. HIF-1α interacts with co-activators such as CREB binding protein (CBP)/p300 [42]. Under normoxia, HIF-1α is hydroxylated by prolyl hydroxylase enzymes (PHD), marking it for binding to an E3 ubiquitin ligase called von Hippel-Lindau (VHL), which adds ubiquitin and allows for proteasomal degradation of HIF-1α [42]. On the other hand, under hypoxic conditions, HIF-1α interacts with p53, which recruits the mouse double minute 2 homolog (MDM2) E3 ligase to degrade HIF-1α [42].

Surprisingly, despite the wealth of information supporting the role of HIF-1α in hypoxia, its isoform, HIF-2α, is also related to physiological processes in PCa. HIF-2α is a protein that shares 48% sequence homology with HIF-1α. The HIF-2α isoform regulates androgen synthesis through another regulator, hypoxia-induced three beta HSD1, which is a reoxygenation cofactor in PCa. This characteristic was therapeutically targeted with the small-molecule PT2399, an inhibitor of the isoform, which decreased PCa proliferation [97] (See Table 3).

An interesting yet controversial aspect of HIF-1α relates to zinc. This assertion may seem disconnected at first glance. However, a study demonstrates the modulation of zinc treatment resistance for PCa, which includes, among various factors, HIF-1α expression, but mainly the activation of the KRAS/PI3K/NF-kB pathway [136]. The discussion about the potential use of zinc as a metabolic treatment for PCa is therefore met with resistance among many researchers, and epidemiological findings are contradictory, as dietary modulation affects zinc availability in the prostate [19,137]. In fact, there is an apparent adaptability to zinc replacement therapy in PCa, which is even multifactorial and context-dependent in the cellular response to eventual treatment. Previously in Table 1, we discussed epigenetic modulation through 5-azacitidine, a DNA methyltransferase (DNMT) inhibitor, in ZIP1 and ZIP3 for PCa. Despite being a particularly exploitable metabolic pathway that would increase zinc availability at the cellular level, it proves to be an unfavorable mechanism. Preclinical results with other nucleoside analogs as DNMT inhibitors for PCa yielded modest results and severe side effects [138], so this therapeutic approach requires careful consideration.

The link between glucose and HIF-1α has been previously described. HIF-1α directly regulates the glycine decarboxylase (GLDC) and is downstream regulated by LDHA in PCa. This marker promotes migration and invasion in PCa. High GLDC activity is associated with high glycolytic activity, high lactate production, and lactate dehydrogenase (LDH) activity [139].

Chromodomain-helicase-DNA-binding protein 1 (CHD1) deletion is among the most common mutations in PCa. CHD1 deletion decreases the expression of prolyl hydroxylase domain protein 2 (PHD2), which catalyzes the hydroxylation of HIF-1α for its degradation by the VHL, thereby increasing glycolysis and angiogenesis in PCa [140]

The joint action of AR and HIF-1α is related to increased synthesis of glycolytic enzymes such as HK2, PFKFB3, and SLC2A1 [141]. Confirmatively, a high rate of HIF-1α expression related to increased HK2 expression has been found in PCa patient cohorts [142].

Despite the high association between HIF-1α and glycolytic metabolism, this transcription factor is also related to lipid metabolism. The mTOR/HIF-1α/MMP2 axis partially constitutes a regulatory lipid metabolism axis during CRPC [143]. There is also a relationship between the HIF-1α/MMP14 pathway, which increases tumor invasion. This activated pathway appears to be increased by adipocytes surrounding the tumor, which is further increased by physiological conditions such as obesity, a risk factor for aggressive PCa. Activation of the pathway occurs through NOX5, an isoform of NADPH oxidase that increases cellular ROS, activating the HIF-1α/MMP14 pathway [144]. At least indirectly, the high availability of lipids activates HIF-1α and aggressive phenotypes of PCa.

An increasing array of substances that inhibit HIF1α, encompassing chemical inhibitors (topotecan, PX-478, YC-1, 2-ME2, BAY87-2243, and digoxin) as well as antisense oligonucleotides (EZN-2968), have demonstrated promising anti-tumor effects by impeding tumor growth and metastasis across various preclinical models [145,146]. Nevertheless, the effectiveness observed in preclinical studies has not been replicated in clinical trials, and the reasons behind this lack of efficacy remain unclear [145,146].

One possibility to counteract the therapeutic failure of HIF1α inhibitors could be found in combinations. The control of HIF1α and hypoxia-induced mechanisms affect the oncogenic protein ID1, increasing its degradation. ID1 functions during HIF1α inhibition, reprogramming PCa to produce GLS2 and enhance glutamine metabolism [146]. ID1 could be a therapeutic target, as it changes the glucose dependence of HIF1α-inhibited cells to glutamine-dependent ones. Dual inhibition of these targets would be a significant therapeutic opportunity, and it would be interesting to observe the molecular mechanism and whether there are other compensatory mechanisms in HIF1α inhibitor-resistant PCa.

### 3.5. Nrf2

The nuclear factor erythroid 2-related factor 2 (Nrf2) is a transcription factor responsible for antioxidant response and cellular detoxification. The activation mechanism of Nrf2 involves its release from an inhibitory protein called Keap1 (Kelch-like ECH-associated protein 1), which is found in the cytoplasm [147]. Keap1 acts as a stress sensor, and under normal conditions, it keeps Nrf2 inactive by directing it toward proteasomal degradation [42,147]. When there is oxidative stress or toxins, specific cysteine residues in Keap1 are modified, altering its ability to retain Nrf2 [42,147]. As a result, Nrf2 is released and translocates to the cell nucleus, where it binds to the promoter region of genes encoding antioxidant and detoxification enzymes through the antioxidant response element (ARE), promoting their transcription [42,147].

Beyond its role in cancer, Nrf2 also plays a crucial role in several non-cancerous diseases due to its involvement in metabolic regulation, inflammation, and autophagy. Its protective function counteracts oxidative stress and inflammation in chronic inflammatory diseases [148] and neurodegenerative conditions. Nrf2 downregulation has been observed in metabolic disorders such as diabetes and obesity [149], where its impairment exacerbates oxidative damage, leading to complications like diabetic nephropathy and cardiovascular disease. Similarly, in neurodegenerative disorders, Nrf2 activation has been proposed as a therapeutic target to mitigate oxidative stress-induced neuronal damage [150]. Furthermore, in inflammatory diseases such as periodontitis [151], reduced Nrf2 expression contributes to increased oxidative stress and worsened inflammation, suggesting that its modulation could be a therapeutic strategy. While Nrf2 activation offers protective benefits in many non-cancerous diseases, its role is complex and context-dependent. In some cases, prolonged or excessive activation of Nrf2 can lead to adverse effects, such as promoting cancer cell survival and drug resistance [152].

This broader role of Nrf2 in maintaining cellular homeostasis extends into cancer metabolism, particularly in PCa. Nrf2 plays a pivotal role in glycolytic reprogramming, lipid metabolism, and androgen biosynthesis, thereby influencing tumor progression and therapy resistance.

In PCa metabolism, Nrf2 has a well-established connection with glycolysis. Through the highly expressed glycoprotein stanniocalcin 1 (STC1) in advanced PCa independent of androgens, an Nrf2-inducing mechanism is achieved that increases glycolysis, enhancing the expression of enzymes such as HK2 and LDHA, as well as other Nrf2 downstream targets, including GPX4, the glutamine transporter SLC7A11, and NQO1 and HO-1 products [153]. Another regulator for Nrf2 is the LINC01116 long non-coding RNA (lncRNA), overexpressed in PCa, which is related to the modulation of autophagy response, cell cycle, and glycolysis (GAPDH). This finding was made through the activation mechanism of the isothiocyanate sulforaphane, a well-known phytotherapeutic compound that induces Nrf2 [154] (See Table 3).

On the other hand, the relationship between Nrf2 and lipid metabolism has also been evidenced in PCa. The expression of the enzyme aldo-keto reductase 1C3 (AKR1C3) and the anabolic pathway of testosterone and dihydrotestosterone synthesis (steroidogenesis) is mediated by Nrf2 in PCa [155]. In the same context, a study found that arachidonic acid and its specific metabolite 5-hydroxyeicosatetraenoic acid (5-HETE) regulate androgen metabolism. Arachidonic acid is metabolized to 5-HETE and reduces androgens by inducing aldo-keto reductase (AKR) family members AKR1C2 and AKR1C3 expression in the human prostate, which in turn is activated via Nrf2 [155]. The correlation between Nrf2 and lipid metabolism may be context-dependent and subject to the type of cancer. Typically, Nrf2 activation is associated with the downregulation of fatty acid synthesis. SFN as an activator induced low expression of ACC1 and FASN in both androgen-dependent and independent PCa. CPT1A also showed low expression levels, which was an unexpected effect [99].

### 3.6. PPARγ

Lipid metabolism is one of the most critical processes for PCa progression and metastasis, with SREBP-1 being the pivotal transcription factor that reprograms tumor metabolism [42]. The PPAR family of nuclear receptors also emerges as another transcription factor essential to tumor lipid metabolism [42]. There are three subtypes of PPARs, α, β (also known as δ), and γ, and their roles have yet to be elucidated regarding PCa progression. However, one of them, PPARγ, can be an exciting research candidate. Evidence of the intracellular signaling processes defined by PPARγ under overexpression models has pointed PPARγ being highly correlated with PCa tumorigenesis, increased lipid metabolism, and modulation under a PPARγ- PGC1α (its coactivator) of the AKT activation [156]. Research has produced promising results, suggesting that PPARγ could be a druggable transcription factor for PCa. The small-molecule T0070907 showed growth inhibition of PCa cells in vitro depending on PPARγ signaling, leading to ACC, AR and FASN downregulation [102].

Another compound with activity on PPARγ is warfarin, an inhibitor of vitamin K epoxide reductase and, therefore, of the vitamin K cycle. Mice treated with warfarin showed inhibition of PPARγ and a low regulation of AR target genes in the prostate, which RNA-Seq could observe [103]. The warfarin strategy, at sub-lethal doses, and with some cautions could represent a viable alternative for chemoprevention of CRPC, as PPARγ is a crucial transcription factor in the steroidogenesis of this aggressive type of PCa [157]. Activation of cholesterol efflux would reduce steroid synthesis in tumor cells. The PPARγ-LXRα-ABCA1 pathway, described by peroxisome proliferator-activated receptor (PPARγ)/liver X receptor (LXR)α/ATP-binding cassette subfamily A member 1 (ABCA1), is a clear example of pharmacological exploitation. This signaling pathway mediates reverse cholesterol transport, especially in macrophages. Once activated by oxidized low-density lipoproteins (ox-LDL), the transcription factors PPARγ and LXRα initiate the transcription of membrane ATP-binding cassette transporters such as ABCA1, which increase cholesterol efflux at the cellular level [158]. Similarly, a previous study found synergistic action of the carotenoid lycopene and T0901317, an LXRα inhibitor [104]. The combined action of the compounds led to a decrease in the proliferation of DU-145 cells by increasing cholesterol released into the medium and an increase in signaling via PPARγ-LXRα-ABCA1 [104].

Other drugs used for their inhibitory action on PPARγ are thiazolidinediones (TZDs). Interestingly, specific findings in patients and meta-analyses suggested that long-term treatment with TZDs might be related to a low incidence of PCa, a hypothesis reinforced by another study showing improvement in symptoms in diabetic patients with PCa undergoing a combined therapy regimen with TZDs and metformin [156]. It was then logical to think that TZDs could be therapeutic strategies for PCa through a PPARγ-dependent inhibition mechanism. However, the mechanisms found behind the antiproliferative action of the TZD known as troglitazone on PCa cells were PPARγ independent. Previous studies suggest that the reduction in PCa growth is due to a reduction in c-MYC expression and extracellular activation of ERK-regulated kinase phosphorylation [159,160].

## 4. The Role of Signaling Pathways as Metabolic Activators

We have observed the importance of the PI3K/AKT/mTOR signaling pathway and its central role in many metabolic pathways. Growth factors such as insulin-like growth factor 1 (IGF-1) and epidermal growth factor (EGF) activate the PI3K/AKT/mTOR pathway antagonized by PTEN. Activation of PI3K/AKT/mTOR and its signaling changes metabolic production from OXPHOS to glycolysis. PI3K/AKT/mTOR also increases biosynthetic programs by activating many of the mentioned transcription factors. For this reason, the pathway, particularly mTOR, is known as a metabolic activator.

Another metabolic activator is AMP activated protein kinase (AMPK), which is a master regulator of metabolic responses [161]. Generally, AMPK promotes ATP-generating catabolic metabolism and, in parallel, prevents ATP-depleting anabolic processes [31]. In the case of PCa, AR induction of OXPHOS in PCa is enterally promoted by AMPK signaling [162]. Due to the importance of this regulator, AMPK has been targeted for therapy. Table 3 summarizes some research approaches employed using inhibition of AMPK signaling [105,106].

One compound, metformin, has been proposed as a repurposing candidate. A previous review highlights the potential of metformin in PCa treatment [163]. There is substantial evidence supporting a transcriptomic response to metformin when used as monotherapy. Though the precise mechanisms by which metformin operates are not fully elucidated, its potential as an anticancer agent is suggested by its direct ability to trigger AMPK and Liver kinase B1 (LKB1), suppression of mTOR activity and protein synthesis, stimulation of apoptosis and autophagy by p53 and p21, reduction in blood insulin levels, and hindering epithelial–mesenchymal transition [30,163,164]. Beyond PCa, metformin has demonstrated anticancer activity across multiple malignancies, including breast [165], colorectal [166], and lung cancers [167]. These effects are primarily attributed to its role in energy metabolism, disrupting cancer cell proliferation by reducing systemic insulin levels, activating AMPK, and inhibiting mTOR signaling.

In the context of PCa, clinical applications of metformin for PCa have shown contradictory results. However, its biological effects might be potentiated in combination with other androgen-targeted agents such as enzalutamide and abiraterone for mCRPC [30] or even in combination with other non-androgen-targeting drugs [163].

The pivotal role of other important signaling cascades can be related to the transcription factor MYC in PCa. Alongside PI3K–AKT–mTOR, the RAS–RAF–MEK–ERK signaling pathway can induce the transcriptional reprogramming of MYC and other transcription factors [168]. Additionally, the Wnt/β-Catenin and JAK/STAT pathways can enhance MYC gene expression [169,170].

## 5. Multi-Target Compounds: Therapeutic Implications of Transcriptional and Epigenetic Targeting in Metabolic Reprogramming of Prostate Cancer

Transcription factors were once an undruggable target due to the complex protein–protein interactions with their co-activators and co-repressors and their specific DNA binding. Different from classical enzyme inhibition, the transcription factor DNA binding sites are charged, and co-activator interaction is much flatter than enzyme ligand binding [171]. Nowadays, many compounds exist, taking advantage of four different transcriptional processes: modulation of epigenetic mechanisms, repurposing the ubiquitin-proteasome system, targeting the molecular chaperone network, and targeting the transcriptional complex [171,172]. In two recent reviews focused on PCa, many small molecules and some specialized compounds are considered promising therapeutic alternatives [171,173]. This type of literature evidence highlights the importance of complex transcriptional regulation in PCa biology, particularly the groundbreaking role of discovered transcriptomic/epigenetic-directed therapies.

In the context of PCa metabolic reprogramming, it is viable that compounds modulating transcriptional processes may disrupt PCa metabolism. These may include inhibitors of transcriptional coactivators P300/CBP (which interact with AR, p53, and MYC), bromodomain extra-terminal (BET) inhibitors, E3 ligase disruptors such as proteolysis targeting chimeras (PROTACs), or even small molecule inhibitors of heat shock 90 kDa (HSP90) chaperone protein [171,173]. Nevertheless, many of these cancer drug candidates, which are transcriptome-directed, tend to be overly specific to non-metabolic pathways. This could be due to two situations: firstly, most compounds are designed to target conventional cancer-related mechanisms, such as inducing apoptosis or inhibiting cell proliferation [174]. Secondly, there is a lack of comprehensive studies to explain how these compounds can impact PCa metabolism.

For example, despite all knowledge, only a few of the small-molecule candidates for direct/indirect targeting of transcription factors serve to elicit metabolic reprogramming modulation in PCa. The ERG oncogene makes part of the TMPRSS2-ERG fusion, typically found in most PCa cancers [175]. ETV1 is part of the aggressive phenotype by running the androgen metabolism in PCa. The small molecules YK-4-279, DB1255, and BRD32048, which acts as inhibitors of the Ets family of gene fusion transcription factors hold promise for PCa treatment. YK-4-279 is a double inhibitor of ERG and ETV1, whereas DB1255 and BRD32048 are ERG and ETV1 inhibitors, respectively [176,177,178]. Given the limited number of these molecules that effectively modulate metabolic pathways, there is a pressing need to explore additional therapeutic avenues.

In pursuit of these avenues, our literature review identified multi-target compounds that can affect multiple enzymes and metabolites essential for modulating PCa processes. These compounds represent a promising category for inducing broader metabolic reprogramming in PCa. Detailed in Table 4, the list includes each compound’s 2D structure, biological data summary and main metabolic reprogramming effects in PCa.

Even though these multi-target compounds have high potential, achieving a complete reversal of multiple phenotypic responses at the cancer level is still challenging. PCa, a heterogeneous disease, exhibits high complexity in the processes that drive its inherent metabolic reprogramming. Nonetheless, multi-target compounds offer a more promising therapeutic option. Their biological action allows them to influence the activity of many enzymes crucial for the progression of PCa almost extensively. These enzymes are mostly related to glucose and lipid metabolism. However, to understand how these compounds work, there needs to be a first central axis deeply linked to the metabolic response, and that axis is likely to be transcriptomic, but with certain epigenetic modulation.

A viable option to start identifying potential candidates for the “phenotypic reversal” of PCa is to analyze compounds with chemopreventive capacity. Most of these compounds are natural products or their derivatives. An example is β-sitosterol, a phytosterol [179] that acts as an aromatase and 5α-reductase inhibitor, and chemopreventive agent against prostate hyperplasia [180]. Evidence of the chemopreventive effects of multi-target compounds comes from previous studies showing a slowdown or decrease in the incidence of PCa. These compounds also act on the epigenetic complex of this cancer. The multi-target compounds described in Table 4 have a naturally occurring origin as one of their main characteristics. Most of these multi-target compounds primarily come from dietary sources (nutraceuticals), plant material, microorganisms, or even marine organisms, offering advantages such as multi-targeted effects and pleiotropic action mechanisms [181].

**Table 4:** Chemical compounds that showed multi-target effects influencing metabolic reprogramming in PCa according to literature. Molecules with * are naturally occurring compounds. When a ** accompanies a chemical name, it refers to an existing synthetized drug, designed or proposed. The † symbol represents a natural product with a pharmaceutical formulation. The chemical structure and clinical trials information were obtained from DrugBank and PubChem databases.

Molecule Name and Specific Chemical Type	Chemical Structure	In Vitro Data	In Vivo Data	Multi-Target Effects
Ursolic acid * (pentacyclic triterpenoid)	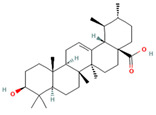 DB: 15588 CID: 64945	IC50: 35 μM (PC3), 47 μM (LNCaP), and 80 μM (DU145) at 24 h [182]	Reduction in tumor growth (VCaP) with 0.1% diet (*w*/*w*) [109]	SAM induction and effects of extracellular matrix remodeling, angiogenesis and cell adhesion in PCa. Epigenetic modification in PCa by modulating PTEN suppressive response
Evodiamine * (indolic alkaloid)	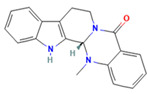 DB: - CID: 442088	Cell viability of 50% with 10 μM (PC3 and DU145) at 48 h [183]	Decreased tumor weight with 20 mg/kg dose (PC3) [184]	Increased expression of semaphorin 3A, and repression of HIF-1 α, H3K18la, GPX4 and PD-L1
Genistein * (isoflavone)	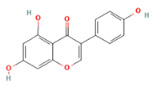 DB: 01645 CID: 5280961	IC50: 15.1 μM (LNCaP), and 35.3 μM (PC3) at 24 h [56]	Tumor reduction with 250 mg/kg diet (high dose) [185]	GLUT and DNMT inhibitor. Epigenetic effects include demethylation of promoter region of metabolism-related tumor suppressor genes such as RAR β, and O6-methylguanine methyltransferase
Juglone * (1,4-naftoquinone)	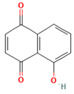 DB: - CID: 3806	IC50: 32.2 μM (LNCaP) at 24 h [186]		HK, PFK, PK, and OXPHOS activity inhibition
β-elemene *† (sesquiterpene)	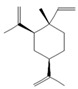 DB: 18097 CID: 6918391	For elemene *† IC50: 146.8 μM (LNCaP) and 215.3 μM (PC3) For β-elemene IC50: 342.5 μM (LNCaP) and 318.1 μM (PC3)		p53 and FZR1upregulation Downregulation of PFKFB3
Tannic acid * (polyphenol)	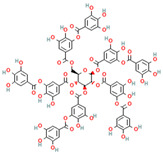 DB: 09372 CID: 16129878	IC50: 29.1 μM (LNCaP), and 35.3 μM (PC3) [187] Decreased cell viability of ~70% with 10 μM at 24 h, and ~50% with 20 μM at 48 h, for C4-2, DU145, and PC3 [67]		Inhibitory effect on ACSL1, ACC, ACeCS1, FASN, Lipin 1
Withaferin A * (withanolide, naturally occurring steroid derived from ergostane skeleton)	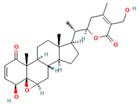 DB: - CID: 265237	Cell proliferation below 50% at 48 h with 1 and 2 μM (LNCaP and 22Rv1) [68]	~67% decrease in carcinoma in situ with 0.1 mg/mouse dose [68]	Inhibitory effect on ACC, ACLY, FASN, CPT1A
Sulforaphane * (isothiocyanate)	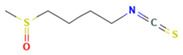 DB: 12422 CID: 5350	IC50: 10 μM (DU145) at 24 h [188]; 40 to 60% cell viability (DU145 and PC3) following 30 μM for 72 h [189]; 10 μM (LNCaP) reverse high AR expression [190]	Slower tumor development rate with high sulforaphane diet (15% broccoli sprouts) [191]; 6 μmol/mouse dose increase 60–70% downregulation of lipid metabolism related enzymes [99]	c-Myc suppression Nrf2 activation Lowered expression of HK2, PK2, LDHA. Lowered expression of ACC, FASN, and CPT1A
T0070907 ** (2-Chloro-5-nitro-N-4-pyridinylbenzamide)	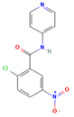 DB: - CID: 2777391	Increased citotoxicity at 100 nM (PC3, LNCaP with high AR and PPARγ and LAPC4) [102]	15 mg/kg dose reduce volume of high AR and PPARγ expression LNCaP xenographs [102]	Impaired PPARγ activity Decreased ACC, AR, and FASN expression

AR: androgen receptor; DB: DrugBank ID; CID: Compound IDentifier PubChem. PFKFB3: 6-phosphofructo-2-kinase/fructose-2,6-biphosphatase 3; FZR1: Fizzy-related protein 1 (or CDC20 homolog 1); ASCL1: achaete-scute homolog 1; ACC: acetyl CoA carboxylase; ACeCS1: acetyl-CoA synthetase; FASN: fatty acid synthase; ACC: acetyl- CoA carboxylase; ACLY: ATP-citrate lyase; GLUTs: glucose transporters; RAR β: retinoic acid receptor beta; H3K18la: specific modification of the histone protein H3, where lysine 18 (K18) is acetylated, GPX4: glutathione peroxidase 4, PD-L1: programmed death-ligand 1; HK2: hexoquinase 2, PK2: pyruvate kinase 2, LDHA: lactate dehydrogenase A; OXPHOS: oxidative phosphorylation; SAM: S-adenosyl-methionine; *w*/*w*: weight/weight, or concentration indicating grams of compound per 100 g of total mixture weight. *† Elemene is an injectable emulsion and class II anti-tumor drug composed primarily of β-elemene, and other compounds such as γ-elemene, and δ-elemene.

Building on the success of naturally occurring multi-target compounds, attention has now turned towards targeting glycolytic metabolism in cancer. The current research trend focuses on altering the activity and expression of GLUTs involving what is known as pan-GLUT inhibitors [192]. The availability of small molecules like DRB-18 or BAY-876, the presence of antisense cDNA therapy, anti-GLUT antibodies, and even naturally derived pan-GLUT inhibitors appears to be the current direction [192], requiring further investigation and application in the context of PCa progression. Nevertheless, the importance of natural compounds is undeniable. In our literature review, a large body of evidence demonstrates the effective multi-target action of both phytochemicals and compounds derived from marine organisms [71]. A clear example is the flavonoids apigenin and phloretin, which modulated GLUT1 and GLUT4 expression, reducing cell proliferation in androgen-independent PCa cells [56] (Table 1).

Natural compounds exhibit interesting activities for enzymes downstream of GLUT activity in the glycolytic process. Here is where multi-target effects mostly occur. The naphthoquinone derivative juglone, an allelopathic compound derived from the Juglandaceae family (particularly the black walnut), which inhibits the growth of other plants, could constitute an alternative treatment against cancer [193]. In the case of PCa, juglone and its multi-target effect inhibited the activity of glycolytic enzymes HK, fructose-6-phosphate-1 kinase (PFK), and pyruvate kinase (PK), consequently inhibiting OXPHOS activity and affecting PCa cell growth [194] (Table 4). The latter might constitute a unique therapeutic effect of juglone as a multi-target compound by inhibiting glycolysis and mitochondrial respiration in AR-dependent PCa cell lines. Nevertheless, inhibition of glycolytic PFK and PK enzymes was also at the mRNA expression level, showing a transcriptomic control. In contrast, HK regulation must follow a possible AR-independent regulation, which remains to be solved in future studies.

More evidence of the therapeutic implications of multi-targeting glycolysis in PCa comes from a recently published work focused on elemene, an anticancer sesquiterpene extract obtained from the rhizome of *Curcuma wenyujin*, which is composed mainly of β-elemene, and other elemene isomers [85] (see Table 3 and Table 4). Validation of β-elemene effects showed a marked downregulation of 6-phosphofructo-2-kinase/fructose-2,6-biphosphatase 3 (PFKFB3), a key activator of the glycolytic rate-limiting enzyme PFK. The latter generated a low glycolytic phenotype in PCa cells. The low expression of PFKFB3 following β-elemene administration was related to the upregulation of p53 and the E3 ubiquitin ligase anaphase-promoting complex/fizzy related protein 1 (APC/FZR1 complex). The binding of p53 to the promoter region of PFKFB3 exerts suppressive control, whereas APC/FZR1 complex degrades PFKFB3, indicating an additional transcriptomic-induced enzyme regulation mediated by β-elemene. The subsequent low glucose intake, and disrupted glycolysis in PCa cells by β-elemene followed multi-target affectations in glutamine metabolism-related enzymes such as glutamine dehydrogenase 1 (GLUD1) and GLS, or glucose-6-phosphate dehydrogenase (G6PD), which is a pivotal for pentose phosphate pathway (PPP) regulation. This “transversality” of β-elemene treatment permeates several metabolic pathways, even PPP, an essential pathway for nucleotide synthesis. Correlated with the later findings, metabolomic results from the juglone study were similar in this fashion since juglone suppressed glycolysis, lipid metabolism, amino acid synthesis, and especially glutamine biosynthesis. How these multiple metabolic disruptions fully operate remains elusive to our current knowledge. Juglone’s ability to influence multiple metabolic pathways could be beneficial in reducing interactions between cellular pathways and minimizing compensatory feedback mechanisms [193].

In addition to the cases described in this review, other promising compounds include tannic acid, a polyphenol known for its ability to modulate lipid metabolism enzyme activity [67]. Tannic acid is a FASN inhibitor, and by disrupting lipogenesis, it affects PCa cellular survival by affecting nuclear and cellular membranes. The lipogenic disruption was accompanied by intracellular variation in gene expression of proteins related to the lipogenic pathway. The tannic acid multi-target effect included the reduction in lipid droplets in vitro, which validated the obtained experimental results.

Another important enzyme for lipid metabolism is ACC. Soraphen A, a natural macrocyclic polyketide originally isolated from the myxobacterium *Sorangium cellulosum*, possesses ACC inhibitory activity [62] (Table 1). Soraphen A decreases the cellular fatty acid supply, by diminishing malonyl-CoA levels, and affecting phospholipid levels, essential to PCa membrane generation. Similar effects of Soraphen A have been encountered in produced drug candidates such as the spiroketone derivative ACC1/2 inhibitor, PF-05175157, developed by Pfizer, and used for type 2 diabetes and nonalcoholic hepatic steatosis. PF-05175157 showed promising results for reducing epithelial cell proliferation in patient-derived prostate explants [63].

Unlike Soraphen A, one compound has multi-target effects in PCa lipid metabolism by using the same principle of regulating an essential metabolite such as acetyl CoA. That is the case of Withaferin A, a naturally isolated steroidal lactone (withanolide) that can be found in *Iochroma gesnerioides*, *Withania coagulans*, *Withania somnifera*, and other members of the *Solanaceae* family. A recent paper shows that Withaferin A can inhibit both ACLY and CPT1A enzymes, affecting both fatty acid synthesis and β-oxidation, diminishing free fatty acid levels in PCa cells. These results were validated in vivo, using transgenic mice with high-Myc phenotype [68] (Table 4). The diminished levels of free fatty acids in PCa cells correlated with low acetyl CoA levels and the induced apoptosis by Withaferin A treatment, suggesting a role of MYC in the inhibition of fatty acid synthesis, which was confirmed by the low expression of other enzymes of de novo lipid synthesis in vivo, such as ACC1 and FASN.

The most recent studies suggest that multi-target compounds disrupt the metabolic reprogramming of PCa by inhibiting specific catabolic or anabolic enzymes and other metabolite-producing enzymes. This inhibition can occur directly to the enzyme or indirectly by interfering with transcriptomic processes. In the case of PCa, a type of cancer that relies primarily on glucose or lipids during certain stages of progression, multi-targeting several key transversal transcriptomic processes or rate-limiting enzyme components of glycolysis, lipid oxidation, or lipid synthesis could have a more pronounced ripple effect on other signaling components related to multiple metabolic pathways. These characteristics present alternatives to disrupt the inherent metabolic reprogramming of PCa.

## 6. Targeting Metabolic Reprogramming and Epigenetics in Prostate Cancer and Therapeutic Opportunities

A recent review showed an exciting concept of crosstalk between metabolic reprogramming and epigenetics in cancer [195]. One of the main ideas of the text lies in the fact that such crosstalk operates in two ways: on the one hand, metabolic reprogramming can modulate cancer epigenetics by providing substrates, cofactors, agonists, or antagonists for epigenetic modifiers and chromatin remodelers. On the other hand, epigenetic mechanisms are involved in cancer metabolic reprogramming by regulating the expression and function of metabolic enzymes and upstream regulators [195].

The epigenetic mechanisms of cancer include DNA methylation, histone modifications, chromatin remodeling, and non-coding RNAs (ncRNAs). Metabolic reprogramming affects cellular epigenetics by regulating the availability of substrates and cofactors for chromatin regulators [195]. Among these substrates are acetyl-CoA and SAM, while as cofactors, we have α-ketoglutarate (α-KG) and NAD^+^.

Acetyl-CoA is a metabolite produced during oxidative decarboxylation, fatty acid oxidation, and amino acid metabolism. During metabolic reprogramming, the ratio of acetyl-CoA to coenzyme A (CoA) may be altered, which could affect the histone acetylation states in cancer cells. In this sense, AMPK, as a regulator of glycolysis and the TCA cycle, acts as a regulator, as seen in some cancers. In the case of PCa, the PI3K/AKT pathway is active, which has been correlated with levels of histone acetylation [196].

The condensation of acetyl-CoA with oxaloacetate to form citrate allows for the availability of acetyl-CoA in the cytosol, where enzymes like ACLY act by controlling acetate metabolism [197]. Many of these acetyl-CoA-producing enzymes are also nuclear. Some spatiotemporal processes, such as DNA damage or other signals, promote the phosphorylation-driven activation of these enzymes, promoting local acetyl-CoA production and generating acetate availability for acetylation [195]. Some of these signals, such as growth factors or mitochondrial dysfunction, increase the pyruvate dehydrogenase complex (PDC) translocation from the mitochondria to the nucleus [195]. For PTEN-deficient tumors, PDC is compartmentalized and located in the nucleus. Nuclear PDC regulates H3K9ac and thus increases the synthesis of lipid metabolism genes through increased expression of SREBP [198].

Acetyl-CoA might have an interconnective role in metabolic reprogramming modulation. Evidence of this comes from studies of sulforaphane treatment on PCa models. Sulforaphane, a bioactive isothiocyanate, derives from glucoraphanin, a glucosinolate isolated from plants of the *Brassicaceae* family. In the arsenal of multi-target compounds, this natural nutraceutical has evidenced multiple effects on diverse metabolic pathways in PCa. Sulforaphane effects on Nrf2 activation were previously described in this review, but further evidence shows sulforaphane administration seems to disrupt both glycolysis and fatty acid synthesis (Table 3 and Table 4) [88,99]. This multiple inference of sulforaphane in critical metabolic pathways in PCa has three potential sources of explanation. Firstly, the upregulation of MYC expression explains the low glycolytic phenotype. Secondly, through the inhibition of SREBP1, explaining the disruption of de novo lipid synthesis. Thirdly, by modulating low acetyl-CoA levels in PCa. In the third case, a hypothesis is that acetyl CoA plays an intercommunicative role between glycolysis and fatty acid synthesis. Is almost logical, since acetyl-CoA, which serves as the building block for fatty acid synthesis, is partly produced through the decarboxylation of the glycolysis intermediate pyruvate, mediated by the pyruvate dehydrogenase complex [88]. By lowering acetyl-CoA levels, sulforaphane might act by disrupting the translocation of acetyl-CoA among metabolic pathways, a mechanism that warrants further investigation.

Another compound that might influence PCa metabolic reprogramming through acetyl-CoA is acetyl-L-carnitine (ALCAR). ALCAR is not an amino acid itself, but the L-carnitine moiety can be found in meat products and some vegetables, such as wheat, soybean and avocado [199]. ALCAR supplementation in PCa shows multi-target effects (Table 1). Such mechanisms involve reducing the activity of pathways like VEGF/VEGFR2 and CXCR4/CXCL12, which are key regulators of angiogenesis and cell migration [66]. This work by Baci and coworkers focused mostly on inflammation, and curiously, works on ALCAR seem scarce in the literature. Therefore, other metabolic reprogramming capacities of ALCAR remain unsolved. Is a hypothesis, but ALCAR might induce acetyl-CoA increase by enhancing the activity of mitochondrial carnitine acetyltransferase, which maintains the carnitine pool to support β-oxidation, producing more acetyl-CoA [200]. This might trigger the epigenetic abilities of acetyl-CoA, which might be interesting to analyze in a comprehensive setting for PCa. In fact, indirect evidence supports the role of ALCAR as a promoter of histone acetylation [201].

Other mechanisms of ALCAR metabolic reprogramming in a multi-target setting might come from the low expression of TNF-α that Baci’s team reported [66]. A recent review summarizes the crosstalk between TNF-α and the canonical activation pathway of NF-κB, a transcription factor that regulates genes involved in inflammation and metabolism [202]. NF-κB activation can lead to increased glucose uptake and glycolysis, promoting a metabolic environment conducive to cancer cell survival and proliferation. The disruption of the TNF-α and NF-κB signaling and its implications for metabolic reprogramming in PCa seems an interesting research area. Other aspects of metabolic regulation, such as AR transcriptomic control of VEGF, remained unsolved in Baci’s work.

Beyond the evidence supporting acetyl-CoA in PCa metabolic reprogramming, another key metabolite plays a crucial role. Previously, we discussed that the DNA methylation profile of prostate adenocarcinoma varies during differentiation. Protein kinase PKCλ/i deficiency increases 1C metabolism through the mTORC1/ATF4/PHGDH axis to enhance DNA methylation, promoting the NEPC phenotype [195,203]. SAM, synthesized from methionine and ATP, constitutes an essential modulator of 1C metabolism. Elevated SAM generation alters the epigenetic landscape of DNA methylation and dynamically supports retrotransposon methylation. SAM is a methyl donor for CpG islands or regions (Cytosine-Guanine sites of DNA sequences) [204]. CpG islands induce histone deacetylation, chromatin remodeling, and gene silencing [204]. Therefore, the epigenetic and post-transcriptional repercussions of SAM modulation are diverse. For this reason, SAM modulation could represent a mechanism that, if correctly exploited, could generate an alternative treatment for PCa.

Recent studies show that treating PCa cells with ursolic acid (UA), a pentacyclic triterpenoid, can modulate SAM [109] (see Table 3 and Table 4). The research demonstrated that UA treatment increased SAM levels in VCaP cells and PCa xenograft implant samples. Researchers employed various sequencing and bioinformatics techniques, using DEGSeq analysis of RNA-Seq data, analysis of differentially methylated regions (DMRs) by Methyl-Seq, and with the assistance of bioinformatic analysis using Metascape, which integrates RNA-Seq and Methyl-Seq data. SAM modulation was associated with clusters found in the Metascape analysis corresponding to canonical pathways affected in PCa samples, such as extracellular matrix restructuring, extracellular matrix organization, positive regulation of cellular mobility, angiogenesis, and positive cell–substrate adhesion relationship [109].

UA, its capacity, and the molecular mechanisms behind its pleiotropic effect in PCa are still under study. However, UA shows promising potential as an alternative treatment or chemoprevention for PCa, as it is a phytochemical found in the peel of apples and pears. In another study, the triterpenoid showed abilities to modulate the suppressive response of PTEN in PCa [205]. UA abrogated the overexpression of differentially expressed genes (DEGs) induced by PTEN deletion of PCa-related oncogenes such as *Has3*, *Cfh*, and *Msx1*, indicating that UA plays a crucial role in PTEN deletion-mediated gene regulation and its potential consequences in cancer interception. Association analysis of DEGs and DMRs identified that the mRNA expression of the tumor suppressor gene *BDH2* and the oncogenes *Ephas*, *Isg15*, and *Nos2* correlated with the CpG promoter methylation status in early-stage comparison groups, suggesting that UA could regulate oncogenes or tumor suppressor genes by modulating their promoter methylation at an early stage of prostate tumorigenesis.

Another of the chromatin regulatory substrates that is arousing interest due to its role in cancer is lactate. This metabolite, a product of the Warburg effect, generates modifications to histones through the process known as lactylation. Despite its importance, the role of lactylation in chromatin modification tends to go unnoticed. However, recent research supports the role of lactylation in PCa and correlates with the transcriptional factor HIF-1α. One of the investigations concluded that HIF-1α lactylation increases the transcription of CEMIP (cell migration-inducing protein), also known as HYBID or KIAA1199, which enables hyaluronic acid binding [206]. KIAA1199 also promotes glycolysis, hypoxia, and vasculogenic mimicry in PCa [206]. Lactate-induced angiogenesis can be harnessed therapeutically. This process was exploited in a model of advanced cancer at the in vivo level with evodiamine (see Table 3 and Table 4), a compound extracted from Fructus Evodiae or *Evodia rutaecarpa* (Juss.) Benth, which increased the expression of Semaphorin 3A (Sema3A), which is poorly expressed in advanced PCa. Sema3A reduced lactylation, and subsequent effects of its high expression repressed HIF-1α, H3K18la, GPX4 (ferroptosis) and PD-L1 [184]. A new opportunity to research hides in elucidating the entire mechanism of lactylation in PCa. Whether this type of hypoxia-related modification can be reproduced with other compounds remains to be discovered.

Another natural compound that has important epigenetic regulation is genistein, an isoflavone extracted from fava beans and soybeans. Not only constitutes a GLUT inhibitor, but an important epigenetic disruptor due to its DNMT inhibition capacities. Epigenetic effects of genistein include demethylation of promoter regions of tumor suppressor genes such as retinoic acid receptor beta (RAR-β), and O6-methylguanine methyltransferase (MGMT) (See Table 3 and Table 4).

Suppression of RAR-β is needed for cell progression, a distinctive trait of metabolic active cancer cells and genistein restricts RAR-β hypermethylation, which is a common feature of other epigenetically active molecules such as dietary polyphenols with the galloyl moiety [207]. The other demethylation process of CpG islands on the promoter region of MGMT gene is another important activity of genistein, since MGMT repression might restrict DNA repair capabilities in PCa cells [208]. The re-activation of RAR-β and MGMT promotes growth inhibition in PCa.

Genistein anticancer effects have been evidenced for several years. It is not unusual that this naturally derived compound was previously directed for clinical studies. Two phase II clinical trials were completed for genistein but showed no consistent results since they failed to recruit enough participants. However, genistein still is a promising therapy for PCa due to its multi-target effects probably by modulating ncRNAs expression and, therefore, affecting metabolic reprogramming. Genistein ncRNA modulation includes the upregulation of miR-34a, miR-573-3p, and miR-1296 [209]. Primarily, miR-34a is a well-characterized tumor suppressor microRNA. Transactivated by p53, miR-34a upregulation correlates with downregulated activity of glycolytic enzymes such as HK1 and LDHA, also reducing lipid synthesis through ACSL1 downregulation in cancer cells [210].

Another factor contributing to the remodeling of the transcriptional process in PCa are long non-coding RNAs (lncRNAs). Genistein takes advantage of modulating oxidative stress and inflammation pathways, which is why the HOX antisense intergenic RNA (HOTAIR), a lncRNA, is another genistein target for inhibition in PCa [211].

The importance of lncRNA in PCa is evident and needs to be taken seriously due to its multiple implications in metabolic reprogramming. The lncRNA PCGEM1 occupies loci in the promoters of metabolic genes related to glucose, lipids, and glutamine metabolism, which overlap with the activity of c-MYC. PCGEM1 promotes the recruitment of c-Myc to its target genes and induces transactivation activities. These findings emphasize the importance of PCGME1 as a crucial transcriptional regulator in the metabolic reprogramming of PCa [212].

**Figure 3:**
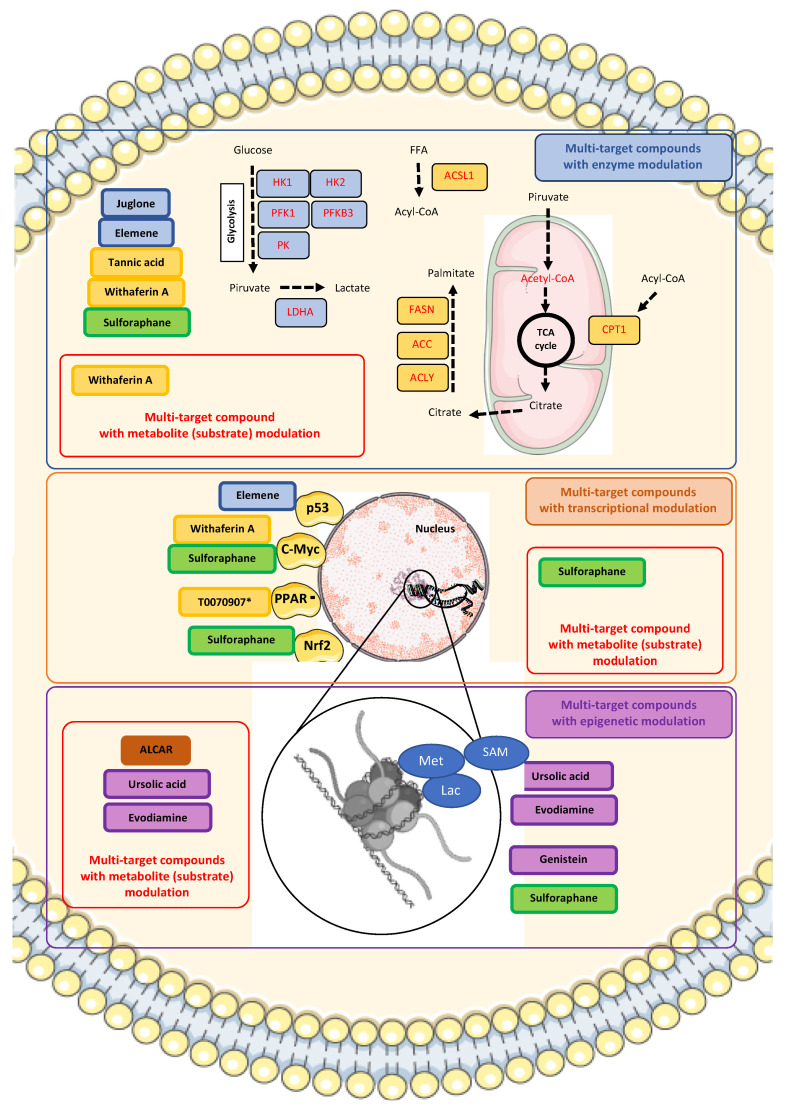
Summary of multi-target promising compounds for prostate cancer (PCa) treatment. Compounds are color-coded based on their predominant metabolic or regulatory activity: blue-labeled compounds act on key enzymes of the glycolytic pathway; yellow-labeled compounds modulate enzymes involved in lipid metabolism; green-labeled compounds exert dual activity by targeting both glycolytic and lipid metabolic pathways. Purple-labeled compounds are linked to epigenetic regulation. Compounds such as ALCAR (brown label) represent substrate-modulating or broad-spectrum agents with multi-target potential, whose mechanisms require further elucidation. Met, histone methylation; Lac, histone lactylation; SAM, S-adenosylmethionine. * Indicates synthetic multi-target compound.

Multi-target compounds are promising for PCa treatment. As summarized in Figure 3, these multi-target therapeutic strategies can be broadly classified into four categories: enzyme modulation, transcriptional modulation, epigenetic modulation, and metabolite-based modulation. These approaches reflect the complexity and interconnectedness of metabolic and regulatory networks in prostate cancer, and underscore the need for comprehensive studies to further explore their therapeutic potential, particularly in the context of protein–protein interactions within the transcriptional landscape.

Based on the current literature, four main categories of multi-target therapeutic strategies can be identified: enzyme modulation, transcriptional modulation, epigenetic modulation, and metabolite-based modulation (including substrate availability or pathway regulation). Comprehensive studies are needed to further validate and expand the therapeutic potential of these compounds. These molecular processes are highly interconnected and may be further refined by considering protein–protein interactions within the transcriptional machinery.

## 7. Clinical Trials

Finally, as a priori analysis about the feasibility of implementing translationally some of the findings described up to here, we have revised clinical trials in prostate cancer (with results) for some molecules discussed above (Table 5). Even though we identified approximately 30 drugs with promising outcomes as modulators of metabolic reprogramming regulators in experimental models, their effectiveness has not yet been tested or demonstrated in the human context for most of them. Safety and sometimes some therapeutic effects have been determined. Compounds like metformin, sulforaphane and lycopene were tested in several studies through different kinds of interventions, evidencing the expectations they have generated among clinicians. Interestingly, most of the drugs shown in Table 5 displayed no serious adverse events, allowing for adjustments in the dosage or interventions that lead to better outcomes. It is expected that this record of clinical trials on prostate cancer with drugs of this type increases soon.

## 8. Key Insights and Perspectives

The importance of metabolic reprogramming in prostate cancer is evident. In recent years, significant advances have been made in elucidating the molecular mechanisms behind this pathology. With the help of omics, the discovery of therapeutic targets is increasing. However, the success of metabolic treatments is limited and only a few candidates are under clinical trials. In addition to the above, there are the characteristics of prostate cancer— a disease of high plasticity modulated by the intrinsic metabolic reprogramming of this type of tumor.

In the face of this scenario, the traditional approach of metabolic inhibition for prostate cancer treatment seems to fall short. Inhibiting a particular metabolic pathway may trigger compensatory mechanisms with another pathway, especially in advanced disease states, promoting resistance phenotypes. Therefore, therapeutic metabolic reprogramming should be viewed from a more holistic perspective. Transcription and epigenetics play a decisive role in shedding light on new molecular mechanisms for prostate cancer treatment, and new therapies.

This review summarizes therapies under research with promissory results for prostate cancer metabolic reprogramming to date, with evidence of transcriptomic modulation. Something is very clear. Epigenetics is necessary due to its profound implications for gene expression. This is how, almost inevitably, a connection is found between these three areas, revealing new molecular biomarkers and therapeutic targets and the need to increase research efforts.

It is remarkable that, through a holistic approach, we can observe how little we know about the substrates for chromatin regulation, their biological effect on prostate cancer, and their impact on the transcriptional response, or whether there is a relationship with other metabolites, such as lactate or α-ketoglutarate to regulate metabolic responses or even alternatives such as modulating cellular immune response. The same global approach from the transcriptomic point of view allows us to see that transcription factor-based therapy is a new line of research that should focus on prostate cancer, and above all, it needs to elucidate the mechanisms behind the epigenetic regulation of transcription factors.

Multi-target compounds offer an interesting therapeutic approach to PCa. Their specific mechanisms, although still under elucidation, seems to be directed to five different processes:

- Enzyme Inhibition: This may occur through direct binding to the enzyme itself or indirectly by modifying allosteric regulation. A compound acting as an inhibitor might also disrupt metabolic signaling by affecting a pivotal enzyme. Evidence suggests that glycolysis, lipid oxidation, and lipogenesis are particularly sensitive to multi-target effects for PCa. Due to their interwoven relationship, transcription factors such as MYC or p53 might be crucial to extend the phenotypic reversal of PCa. Omics approaches, with a focus on metabolic flux analysis, may be required to fully elucidate the specific reprogrammed pathways in the case of phenotypic reversal.
- Direct Transcription Factor Binding: Compounds may directly bind to disrupted transcription factors, altering their conformation and promoting a reversal in metabolic reprogramming response.
- Pathway Modulation: By targeting upstream regulators or interacting pathways, the compound can indirectly affect the activity of transcription factors. For PCa, there is particular interest in acetyl-CoA modulation, which requires further research.
- Epigenetic Changes: The compound could induce epigenetic modifications that influence the accessibility of transcription factors to DNA. This might include affecting the activity of DNMT, modulation of ncRNAs, and, in particular,, SAM regulation, which seems to induce a favorable metabolic reprogramming in PCa and is related to other important processes of tumor progression.
- Protein–Protein Interactions: The compound might disrupt or enhance interactions between transcription factors and their co-factors or inhibitors. More comprehensive studies using omics data are needed to fully understand the correlation with metabolic reprogramming in PCa.

With advances in RNA-Seq technology and other omics, the global trend in cancer treatment is to achieve multiple responses at the level of metabolic reprogramming. For this, phytotherapeutics and their pleiotropic effect are an exciting alternative that deserves global attention towards bioprospecting to favor the discovery of new bioactive molecules or extracts and even promote new drug designing. Although most of the naturally derived compounds shown in Table 4 are promising, pentacyclic triterpenoids and isothiocyanates seem to have an advantage that could favor reversions to more favorable phenotypes of prostate cancer.

Along with UA, other natural pentacyclic triterpenoids are emerging as promising compounds for therapeutic alternatives in PCa. Nummularic acid (NA) and 3-epifriedelinol (EFD), derived from the aerial parts of the medicinal food plant *Ipomoea batatas*, commonly known as sweet potato, represent promising alternatives for PCa treatments [235,236]. NA and EFD modulate a common mechanistic pathway that includes the enhancement of caspase and PARP cleavage and elevation of BAX and P53 expression, followed by a decrease in BCL-2 and NF-κB expression, thereby triggering apoptosis in PCa cells. These studies align with substantial evidence in the literature that highlights the importance of the ursane skeleton, a primary structural component of ursane saponins, as pharmacologically relevant compounds for PCa treatment.

The same principle as NA and EFD applies for sulforaphane-like molecules. The isothiocyanate (ITC) moiety is an essential structural component present in molecules with antiproliferative activity against PCa [237]. An example is the pterostilbene isothiocyanate conjugate (PTER-ITC), another AR regulator for PCa. Treatments with PTER-ITC demonstrated IC50 values of 40 μM and 45 μM for LNCaP and PC3 cell lines, respectively [238]. The ITC moiety and conjugates might be interesting in designing more multi-target compounds that can influence both glycolytic and lipid metabolism.

However, despite their promise, one of the aspects to overcome for these multi-target compounds is their poor water solubility, which affects their bioavailability and challenges that must be addressed to enhance their therapeutic effects [179]. As essential enzyme inhibitors or transcriptomic disruptors, where several targets are located in internal cellular compartments, more pharmacokinetic-focused studies are needed to ensure the success of these multi-target compounds.

SAM modulation holds promise as a metabolic reprogramming strategy for prostate cancer (PCa). Compounds that can increase intracellular SAM levels may have an impact on the epigenetic disruption of this type of cancer. A recent study showed that SAM can directly modulate epigenetic processes in PCa by influencing histone methylation. Specifically, treatment with 200 μM SAM led to changes in gene expression related to alterations in histone methylation at H3K4 and H3K27 [239]. This treatment also resulted in reduced cell viability in various PCa cell lines, indicating the biological effects of SAM [240].

The importance of transcriptomic based control in metabolic reprogramming is increasing. It seems that new interconnective correlations have been discovered and PCa is not an exception. An example might be the utilization of PPARγ antagonists for the control of AR transcriptional regulation. In a previous study, it was found in vitro that AR and PPARγ co-localize in the nucleus of LNCaP cells with constitutive expression of these transcription factors [102]. In a subsequent analysis, the study authors concluded through the use of databases and bioinformatics tools that PPARγ regulates AR activity. This highlights the need for more computational studies focused on database mining of promotor sequences and transcriptional motifs, along with ChIP-seq data at GEO (Gene Expression Omnibus) or ENCODE databases, and an experimental validation by ChIP-qPCR. This PPARγ-AR crosstalk might be an interesting multi-target exploitable pathway. In Table 4 we analyze T0070907, the only non-naturally derived compound in the literature review. The mechanism of T0070907 as a PPARγ inhibitor created a multi-target response of de novo lipid synthesis disruption. This might be interesting for other chemical analogs of T0070907 to act directly to this PPARγ-AR regulation.

A critical perspective emerging from the current research landscape is the relative scarcity of studies addressing therapeutic combinations and their pharmacological or therapeutic effects. The quest for synergistic interactions among monotherapy treatments to develop combination therapies stands as a pivotal area of development in research, particularly given the plastic nature of prostate cancer and its high specificity, along with its unique metabolic regulation. Directing studies towards the observation and analysis of transcriptional mechanisms regulated by these combinations is essential. A recent study conducted by Díaz and colleagues [241] observed how the transcriptional signature changes, eliciting novel responses at the level of transcription factors that are entirely independent of monotherapies. This capacity for transcriptional modification through combinations represents a promising alternative deserving further scrutiny through additional research.

One of the mechanisms that most influence prostate cancer progression is de novo fatty acid synthesis and steroidogenesis. According to the literature review conducted in this paper, an optimal metabolic intervention will likely require targeting these two processes simultaneously for maximum efficacy. Therefore, repurposing candidates or new multi-target drugs and more complex models and experiments are needed. Other metabolic pathways requiring further attention are 1C metabolism and glutaminolysis, especially in resistant prostate cancer.

Despite being a rare type of PCa, NEPC tumors with complete AR-null phenotype have risen in incidence in patients with aggressive mCRPC in recent years [31,57]. Small-cell histological characteristics among mCRPC tumors represent a poor prognosis for a PCa patient [55,58]. There are gaps in understanding the underlying tumor biology of NEPC cancers, but several lines of information can be described respecting genomic alterations in NEPC, as previously reviewed [59]. In addition to a decrease in the expression of AR, low levels of prostate-specific antigen (PSA) and prostate-specific membrane antigen (PSMA) are characteristic of NEPC [56].

Metabolic reprogramming, however, requires further investigation since limited evidence is known about which metabolic features rule during NEPC. We previously showed a recent work identifying increased serine biosynthesis following the loss of PKC λ/ι in NEPC progression through DNA methylation [49]. Other metabolic features of NEPC were previously reviewed [31], such as decreased GLUT12 expression but increased glucose uptake and lactate production. Further research is needed to fully address NEPC characteristics, oversee potential therapeutic strategies or clinical biomarkers, and determine whether other metabolic pathways, metabolites, or transcriptional control are involved.

An emerging perspective in designing multi-target therapies for PCa lies in the strategic integration of non-invasive biomarkers—particularly those obtained from liquid biopsies—as dynamic tools for monitoring therapeutic response. Notably, several of these biomarkers possess molecular characteristics tightly connected to the transcriptomic and epigenetic circuits explored throughout this review. For example, PCA3 is a long non-coding RNA transcriptionally regulated by AR signaling and modulated by epigenetic mechanisms such as promoter methylation and histone modification. Likewise, the TMPRSS2:ERG gene fusion, commonly assessed in platforms like MiPS, arises from AR-driven transcriptional activity and reshapes the transcriptomic landscape of prostate tumors. Other assays such as SelectMDx and ConfirmMDx detect DNA hypermethylation patterns in tumor suppressor genes (e.g., GSTP1, RASSF1), which are influenced by the availability of metabolic cofactors like S-adenosylmethionine (SAM) and acetyl-CoA—central intermediates in epigenetic regulation. An extensive review further discusses these biomarkers for PCa [242].

These findings highlight that liquid biopsy biomarkers are not isolated clinical indicators but are intrinsically linked to the regulatory axes targeted by metabolic and epigenetic modulators. Their expression levels may thus serve as functional surrogates to evaluate pharmacodynamic responses to natural multi-target compounds. For instance, downregulation of PCA3 or TMPRSS2:ERG may reflect effective inhibition of AR signaling or chromatin remodeling, while shifts in DNA methylation markers could indicate successful modulation of DNMT or HDAC activity. As discussed in the Clinical Trials section, most clinical parameters in Table 5 for assessing patient outcomes were limited to PSA, excluding other important molecular biomarkers. Therefore, incorporating such liquid biopsy biomarkers into future trial designs represents a promising strategy to enable real-time, non-invasive monitoring of treatment efficacy. This integrative approach may support more agile therapeutic adjustments, promote precision oncology, and expand the translational value of epigenomic and epigenetic-targeted PCa interventions.

This work highlights the importance of naturally occurring compounds and their multi-targeting effects. However, repurposed compounds might be an important source of potential therapies. Comprehensive omics and computational approaches include artificial intelligence-powered drug repurposing [243]. Another essential approach uses omics techniques to create genome-scale metabolic models (GEMs) for repurposing compounds. Among GEM applications, this metabolic-centered model can be directed to test drug candidates that can elicit inhibition of cancer-related phenotypes [244]. For PCa, compounds discovered under this approach included etomoxir, an inhibitor of long-chain fatty acid transport to the mitochondria [245], and ifenprodil, an NMDA receptor antagonist [246]. Results obtained through GEM studies might increase multi-target compound applications and reinforce the pivotal role of metabolic reprogramming targeting for PCa treatment since metabolic changes are crucial to detecting and treating PCa regardless of the disease stage [247]. Most of the methodology to validate GEMs uses drug-induced transcriptomic data to assess gene-drug interactions, reveal drug off-targets, and further evaluate findings by 2D cell viability assays. Data in 3D culture settings, which recapitulate some essential tumor characteristics, such as hypoxic regions, are needed to strengthen GEMs-related data.

Metabolic-directed 3D culture-focused studies are few, especially for prostate cancer. Additionally, the approach of combinations in such in vitro systems mimicking the tumor is limited and needs to be improved due to their lack of reproducibility in the context of combination therapy discovery [248]. The latter is compounded by the physiological differences that would result from applying such models. A clear example occurs in the case of models of PPARγ overexpression. Two-dimensional culture of PC3 cells overexpressing PPARγ gamma did not affect cell growth, but evidence of an EMT phenotype was found in PC3 spheroid culture with overexpressed PPARγ [156].

## 9. Conclusions

Although classical and chemosensitization approaches improve metabolically focused PCa treatment, intricate compensatory pathways can sustain and preserve the inherent metabolic reprogramming of PCa. This requires a more pronounced induced phenotypic reversal. Transcriptomic and epigenetic-directed therapy can have a major influence on metabolic reprogramming processes in PCa. In addition, multi-target compounds, especially naturally derived metabolites, can elicit transversal responses in diverse metabolic pathways, particularly in p53 and MYC transcription factors, generating a more pronounced reprogramming response in enzyme activities and producing partial phenotype reversal and decreased cell viability in PCa.

As shown in this review, extensive evidence from the literature shows that transcriptomic disruption and epigenetic regulation are pivotal mechanisms to essential metabolic pathways in PCa. Multi-target compounds such as juglone, tannic acid, and withaferin A are promising for PCa treatment. However, pentacyclic terpenoids and isothiocyanate-containing moiety molecules need further research as alternatives for more intensive drug design due to their novel anticancer molecular mechanisms, metabolite modulation of SAM and acetyl-CoA, which corresponds to another interesting and novel area for PCa treatment.

In conclusion, the presence of a dynamic cooperative crosstalk between metabolic reprogramming processes, transcription factors, and epigenetics opens up new possibilities in therapy research for prostate cancer. This allows for the elucidation of therapies with the aid of omics that can potentiate chemotherapy research.

## Figures and Tables

**Table 3:** Summary of studies focused on deregulated transcription factors and epigenetic-related modifications with metabolic reprogramming implications in prostate cancer.

Transcription Factor/Signaling Pathway or Metabolic Regulator/Substrate for Chromatin Modification	Type of Intervention	Main Results	Research Phase	Reference
**AR**	1,2-bis(isothiazol-5-yl) disulfane, or NU9056/single treatment	Decreased expression of AR and PSA in PCa cells.	In vitro	[79]
**p53**	Tenovin-1 and BI2536 in combo with metformin/combined treatment	Enhanced inhibition of OXPHOS in PCa cells with inactivated p53 WT. Chemosensitization, particularly in C4-2 cells (CRPC).	In vitro	[80]
	Flubendazole/single treatment	Upregulation of p53 and increased ferroptosis CRPC. Dysregulation of SLC7A11.	In vitro	[81]
	Metformin and 2DG/combined treatment	Increased p53-regulated apoptosis via AMPK.	In vitro	[82]
	Piceatannol/single treatment	No modulation of glucose metabolism, but p53 upregulation.	In vitro, In vivo	[83]
	FR194738/single treatment	Inhibition of SQLE with FR194738 alters CRPC program.	In vitro	[84]
	Beta-elemene from Curcuma wenyujin/single treatment	Glycolysis inhibition in PCa cells by downregulating PFKFB3 expression. Increased p53 and FZR1 expression.	In vitro, In vivo	[85]
	Biomarker focalized study/promissory target: DDR1	Restoration of p53 and DDR1 transcriptomic activity enhances the response to signals that “reverse” the cancerous phenotype in prostate cancer.	In vitro	[86]
**MYC**	Sulforaphane/single treatment	Suppression of c-Myc expression and stem-like characteristics in PCa.	In vitro, In vivo	[87]
		Suppression of c-Myc metabolic activity, lowered expression of HK2, PK2M2 and or LDHA, in models of c-Myc overexpression without affecting plasma lactate levels measured in plasma.	In vitro, In vivo	[88]
	10074-G5/10058-F4 and 6-diazo-5-oxo-L-norleucine/combined treatment	Induction of GFAT1 by MYC inhibitors, with additional GFAT1 inhibition with 6-diazo-5-oxo-L-norleucine showed synergistic effects by inhibiting proliferation of PCa.	In vitro, In vivo	[89]
	ST045849 and alanine aminotransferase inhibitors/combined treatment	OGT and GPT2 inhibitors decrease cell viability and growth of PCa. OGT inhibition leads to c-Myc loss.	In vitro	[90]
	Mycophenolic acid/combined treatment	IMPDH inhibition, combined with antiandrogens, decreases GTP and stabilizes p53.	In vitro	[91]
	Diphenylamines derivatives, compound 7d/single treatment	Compound 7d terminates coregulatory function of BET and AR domain, transactivation of wild-type AR, enzalutamide resistance mutants, downregulation of c-Myc expression by BET inhibition. Terminates VCaP cells with AR-V7 variant in vivo.	In vitro, In vivo	[92]
	1,25-dihydroxyvitamin D-3/single treatment	Low glycolysis, dysregulated TCA, low c-Myc expression.	In vitro	[93]
	Biomarker focalized study/promissory target: OTUD6A	OTUD6A acts as a physiological deubiquitinase for c-Myc, stabilizing it.	In vitro, In vivo	[94]
	Biomarker focalized study/promissory target: HSP90-CIpP	HSP60 regulates CIpP expression via c-Myc, restoring mitochondrial functions in advanced PCa.	In vitro, In vivo	[95]
**HIF-1**	NSC-134754/single treatment	Decreased HIF1α and GLUT1 expression. Low glucose intake in PCa.	In vitro, In vivo	[96]
	PT2399/single treatment	Inhibition of HIF2α. Decreased PCa proliferation.	In vitro	[97]
	Sulforaphane/single treament	Decreased HIF1α nuclear translocation. Increased mitochondrial biogenesis and activity	In vitro	[98]
**Nrf2**	Sulforaphane/single treament	Low expression of ACC1 and FASN in androgen-dependent and androgen-independent PCa. Low CPT1A expression.	In vitro, In vivo	[99]
		Increased Nrf2 nuclear translocation. Increased mitochondrial biogenesis and activity	In vitro	[98]
**FOXO**	Apigenin/single treatment	Decreased tumor size in TRAMP mice model. Loss of FOXO3a phosphorylation generates its activation, by decreasing 14-3-3 binding. Reduced PCa proliferation	In vivo	[100]
	Methyl protodioscin (furostanol saponin)/single treatment	Reduced MAPK signaling activity. Induced FOXO1 expression, which increased apoptosis in PCa model in vivo.	In vitro, In vivo	[101]
**PPAR** **γ**	T0070907/single treatment	Disruption in cell growth by impaired PPARγ signaling, following decreasing ACC, AR, and FASN expression in PCa cell lines	In vitro, In vivo	[102]
	Warfarin/single treatment	PPARγ inhibition and low expression of RA target genes.	In vitro	[103]
	Lycopene and T0901317/combined treatment	Increased PPARγ-LXRα-ABCA1 expression, increased cholesterol eflux, synergistic effect of increased PPARγ-LXRα-ABCA1 following lycopene and T0901317 combined treatment	In vitro	[104]
**AMPK**	MT 63–78 and AR signaling inhibitors/combined treatment	AMPK activation, mTORC blockade, increased growth inhibitory effect of AR signaling inhibitors MDV3100 and abiraterone	In vitro	[105]
	Salicylate and radiotherapy/combined treatment	Salicylate enhanced the effects of radiotherapy on AMPK and ACC but blocked markers of mTOR activation	In vitro, In vivo	[106]
**mTOR**	Arctigenin/single treatment	Inhibition of proliferation, decreased AR, decreased circulating FFAs, IGF-1, VEGF, MCP-1, and increased Nkx3.1	In vitro, In vivo	[107]
**SIRT1**	Astragalus Polysaccharides/single treatment	Decreased SIRT1 expression, decreased proliferation and invasion of PCa cells and decreased cellular triglyceride and cholesterol levels	In vitro, In vivo	[108]
**Acetyl CoA and SAM**	Ursolic acid/single treatment	Increased SAM, Nrf2-mediated oxidative stress response, CXCR4 signaling, TGF-β signaling. CpG methylation sites and anti-metastatic reprogramming	In vitro, In vivo	[109]

AR: androgen receptor; PSA: prostate specific antigen; OXPHOS: oxidative phosphorylation; CRPC: castration resistant prostate cancer; SLC7A11: solute carrier family 7 member 11 (glutamine metabolism); SQLE: squalene epoxidase; PFKFB3: 6-phosphofructo-2-kinase/fructose-2,6-biphosphatase 3; FZR1: Fizzy-related protein 1 (or CDC20 homolog 1); DDR1: discoidin domain receptor; HK2: hexoquinase 2, PKM2: pyruvate kinase M2; LDHA: lactate dehydrogenase; GFAT1: Glutamine:fructose-6-phosphate amidotransferase; OGT: O-Glc-Nac transferase; IMPDH: inosine-5′-monophosphate dehydrogenase; GTP: guanosine triphosphate; BET: Bromodomain and Extra-Terminal motif; AR-V7: androgen receptor splice variant 7; GPT2: alanine aminotransferase; WT: wild-type; OTUD6A: OTU Domain Containing 6A; HSP60: heat shock protein 60; CIpP: caseinolytic protease P; TCA: tricarboxylic acid cycle; GLUT1: glucose transporter 1; ACC1: acetyl carboxylase 1; FASN: fatty acid synthase; CPT1A: carnitine palmitoyl transferase 1 A; RA: retinoic acid; VEGF: vascular endothelial growth factor; FFAs: free fatty acids; SIRT1: sirtuin 1; SAM: s-adenosyl methionine; TGF-β: transforming growth factor beta; IGF-1: insulin growth factor 1; CXCR4: chemokine receptor C-X-C chemokine receptor type 4; CpG: cytosine-guanine methylation island; mTOR: mammalian target of rapamycin; PPARγ–LXRα–ABCA1: Peroxisome proliferator-activated receptor gamma—liver X receptor alpha—ATP-binding cassette transporter A1 axis; AMPK: AMP-activated protein kinase; MCP-1: monocyte chemoattractant protein-1.

**Table 5:** Some clinical trials involving drugs that have yielded preclinical outcomes on dysregulated transcription factors or epigenetic regulators leading to metabolic reprogramming (including multi-target effects) in prostate cancer.

Drug	Trial Registration and/or Reference	Intervention/Treatment	Main Outcomes/Adverse Events
Metformin	NCT01215032	Metformin taken orally twice daily each 28-day cycle, for 12 cycles, in men with CRPC	Lack of efficacy. No serious adverse events
	NCT02614859 [213]	Metformin (1000 mg) twice a day plus bicalutamide (50 mg) daily for 24 weeks in overweight or obese PCa patients	Metformin induced modest PSA declines in 40% of patients after 8 weeks. No serious adverse events.
	NCT01796028 [214]	Docetaxel (75 mg/m^2^) plus metformin (850 mg) oral twice a day on a continuous daily dosing schedule in men with metastatic hormone-refractory PCa	Metformin addition failed to improve the standard regimen
	NCT03137186 [215]	Metformin (850 mg) once or twice daily combined with the standard treatment of locally advanced PCa patients	Combining with ADT, CRPC-free survival was significantly improved with metformin mainly in locally advanced or metastatic PCa. No significant adverse events apart from grade II diarrhea.
	NCT:01620593 [216]	Metformin (500 mg) twice or three times a day after castration for 28 weeks in non-diabetic patients with biochemically relapsed or advanced PCa	Metformin added to ADT did not show differences in PSA response. Adverse events overall were increased in the metformin cohort compared to placebo
Sulforaphane	NCT01228084 [217]	Sulforaphane (200 μmol) daily for 20 weeks in men with recurrent PCa	5% of patients achieved ≥50% decline in PSA. No serious adverse events.
	NCT01265953 [218]	Sulforaphane (200 μmol) daily for 4 weeks in men scheduled for a prostate biopsy	No significant difference in prostate HDAC activity or Ki67. No serious adverse events.
	NCT00946309	Sulforaphane (100 umol), broccoli sprout extract) every other day for 5 weeks in men with low or intermediate-grade PCa	No serious adverse events.
	NCT01950143 [219]	Glucoraphanin-rich broccoli soup weekly for 12 months in men on active surveillance	Attenuation of changes in prostate gene expression and associated oncogenic pathways
Lycopene	[220]	Lycopene (15 mg) twice daily for 3 weeks before radical prostatectomy in patients with a diagnosis of PCa	Decrease in plasma PSA level and tumor volume. No adverse events reported
	[221]	Lycopene (2 mg) twice daily plus orchidectomy in patients confirmed with metastatic PCa	More reliable and consistent decrease in serum PSA level in lycopene patients still alive after 2 years. No adverse reactions
	[222]	Dose-escalating (15–120 mg/day for 1 year) trial of lycopene in patients with biochemical relapse of PCa after definitive local therapy	No serum PSA responses were observed. Lycopene supplementation was safe and well tolerated
	[223]	Lycopene supplementation (15 mg) daily for 6 months in patients with progressive hormone refractory PCa	No clinically relevant benefits were observed
	NCT00416325 [224]	Tomato extract capsules containing lycopene (30 mg) once, twice, or three times daily for 3 months in patients who are at high risk of developing PCa	No treatment effects were apparent on either the serum or benign tissue endpoints
	NCT01882985 [225]	Docetaxel on day 2 and lycopene (30 mg) once daily on days 1–21 for at least 4 courses in chemotherapy-naïve PCa patients	PSA response rate of 76.9% and median survival of 35.1 months versus 45% PSA response rate and 17.4 months median survival reported for docetaxel plus prednisone (TAX 327 trial). No patients experienced grade 3 or above anemia.
1,25-dihydroxyvitamin D3	[226]	Calcitriol (0.5–2.5 µg) daily for 6 to 15 months in patients with recurrence indicated by rising serum PSA levels after primary treatment with radiation or surgery	Rate of PSA rise during significantly decreased in 6 of 7 patients versus before calcitriol therapy. Dose-dependent calciuric side effects
	[227]	Calcitriol (0.5 µg/kg) on day 1 followed by docetaxel (36 mg/m^2^) on day 2, repeated weekly for 6 weeks of an 8-week cycle in patients with metastatic androgen-independent PCa	PSA, time to progression and survival are promising when compared with contemporary phase II studies of single-agent docetaxel.
	[228]	Calcitriol (0.5 µg/kg) on day 1 and carboplatin (AUC 7 or AUC 6 in patients with prior radiation) on day 2, repeated every 4 weeks in patients with metastatic androgen-independent PCa	No increase in the response rate when compared with the activity of carboplatin alone. Toxicity was mild and generally similar to that expected with single-agent carboplatin
	[229]	High-dose, intermittent calcitriol (8 -12 µg) plus dexamethasone 4 mg in patients whom prostate cancer was progressive despite androgen deprivation	Response rate was not found to be clearly higher than expected with dexamethasone alone. Combination appears to be safe
	NCT00741364 [230]	Vitamin D (400 IU, 10 000 IU or 40 000 IU) once per day, for a 3- to 8-week period ending the day before radical prostatectomy	Vitamin D3 modestly lowered PSA and PTH. All reported side effects were classified as grade 1
Genistein	[231]	Genistein-rich extract three times daily for six months in patients with histologically proven PCa	Genistein-rich extract did not reduce PSA levels by 50% or more in 51 of 52 subjects
	NCT00058266 [232]	Genistein (150 mg) once daily for 1–2 months in patients that undergo radical prostatectomy, and then continue oral genistein once daily for 1–2 months afterward (3 months of therapy)	MMP-2 transcript level in normal prostate epithelial cells was higher in the untreated group than in the genistein-treated group
	[233]	Synthetic genistein (30 mg) daily for 3–6 weeks before radical prostatectomy in patients with localized PCa	PSA decreased by 7.8% in the genistein arm and increased by 4.4% in the placebo arm. Adverse events were few and mild.
Warfarin	[234]	All warfarin uses among 78,615 men during 1995–2009 was analyzed and estimated PCa risk overall, and by tumor grade and stage.	A similar risk of PCa was found among warfarin users and the general population
2-Deoxyglucose	NCT00633087	2-deoxyglucose (30 mg/kg) daily for two weeks of a three-week (21 day) cycle in patients with advanced and hormone refractory PCa	75% (9/12) of 5-year progression-free survival; 58.3% (7/12) of 5-year overall survival; 25% (3/12) of serious adverse events

ADT: androgen deprivation therapy; AUC: area under the concentration time curve; CRPC: castration resistant prostate cancer; HDAC: histone deacetylase; MMP: matrix metalloproteinase; PCa: prostate cancer; PSA: prostate specific antigen; PTH: parathyroid hormone.

## Data Availability

Data are contained within this article.
